# The associations between resting and total energy expenditure, physical activity, and thyroid hormone levels in adult females

**DOI:** 10.3389/fphys.2026.1716140

**Published:** 2026-03-31

**Authors:** S.A Sanduni Samudika De Alwis, Xueying Zhang, Huihui Mei, Xinyi Bi, Xinyue Ma, Ying Liu, Li Xue, Dehuang Kong, Lu Wang, Hongbo Wang, John R Speakman

**Affiliations:** 1 Shenzhen Key Laboratory of Metabolic Health, Center for Energy Metabolism and Reproduction, Shenzhen Institutes of Advanced Technology, Chinese Academy of Sciences, Shenzhen, China; 2 University of Chinese Academy of Sciences, Beijing, China; 3 School of Pharmacy, Key Laboratory of Molecular Pharmacology and Drug Evaluation, Ministry of Education, Yantai University, Yantai, China; 4 Health Sciences Institute, China Medical University, Shenyang, Liaoning, China; 5 Institute of Genetics and Developmental Biology, Chinese Academy of Sciences, Beijing, China; 6 School of Biological and Environmental Sciences, University of Aberdeen, Aberdeen, United Kingdom

**Keywords:** body composition, energy expenditure, physical activity, resting metabolic rate, thyroid hormones

## Abstract

**Introduction:**

Physical activity (PA) costs energy. However, recent theory suggests the relationship of increasing PA to total energy expenditure (TEE) is non-linear because high levels of activity suppress resting metabolism mediated via changes in thyroid hormones (THs; T3- triiodothyronine, T4- thyroxine, fT3- free triiodothyronine, fT4 - free thyroxine, TSH - thyroid stimulating hormone).

**Method:**

We conducted a cross-sectional study on 38 euthyroid females (NCT06377943) to examine the relationship between PA, resting metabolic rate (RMR), and TEE. Multiple linear regression and Bootstrapped structural equation model were used to assessed whether THs statistically accounted for the association between PA and RMR.

**Results:**

Bootstrapped structural equation models indicated that PA was significantly associated with lower TH levels (β = –4.33, 95% CI [–8.51, –1.70], p = 0.011), while the association between THs and RMR was small and non-significant (β = 0.043, 95% CI [–0.02, 0.10], p = 0.16), resulting in a non-significant indirect effect (β = –0.19, 95% CI [–0.67, 0.08], p = 0.31). In multiple regression plots, physical activity energy expenditure (PAEE) and PA showed a trend toward significance (p = 0.07) when THs were added.

**Conclusion:**

Overall, although PA was associated with modest changes in THs, these changes did not correspond to any detectable decrease in RMR.

## 1 Introduction

Energy balance, or the dynamic equilibrium between energy intake and expenditure, is a fundamental concept used to understand changes in body weight over time (Hall et al., 2012; Hill et al., 2012). Prolonged disruption of energy balance leads to metabolic syndromes such as obesity and cachexia (Vaitkus et al., 2015). TEE is the total energy expended each day, and it comprises three primary components: RMR, PA, and the thermic effect of food (TEF). Among these components, TEF remains relatively stable (around ~10% of TEE), while RMR and PA are highly variable between individuals. This variability arises from the differences in individual body size (height, weight, body composition), age, sex, and hormonal factors, including variations in thyroid hormone levels (Fukagawa et al., 1990; Arciero et al., 1993; Johnstone et al., 2005; Speakman and Westerterp, 2010; Pontzer et al., 2021; Hu et al., 2022).

PA, normally considered the second-largest component of the TEE, is also correlated with serum TH levels. Previous studies which assessed the relationship between physical activity and thyroid hormone has focused on exercise or sport-based activity predominantly in male athletes and military personnel (Opstad et al., 1984; Pakarinen et al., 1991), leaving females as an underrepresented group in thyroid and physical activity studies. Furthermore, given that females have a higher risk of developing thyroid-related complications, it is critical to understand thyroid-metabolic interactions in females. Although there are studies on the THs’ influence on physical performance and muscle strength (Greco et al., 2023; Greco et al., 2025), evidence on the association between habitual physical activity and thyroid is limited. Among the few studies that have examined the relationship between THs and habitual PA, one recent cohort study indicated no association between thyroid status and PA (Roa Dueñas et al., 2021). Yet survey data from the NHANES database on the physical activity of American adults revealed that men with higher levels of weekly physical activity tended to have lower TSH and lower total T4 levels (Tian et al., 2024). The direction of causality in the relationship between THs and PA remains unclear. One possible explanation for this link is derived from the constrained energy expenditure model, which posits that elevated physical activity suppresses THs, which then reduces RMR to maintain the TEE within a narrow range (Pontzer, 2015; Pontzer, 2017; Pontzer, 2018). However, an equally tenable hypothesis is that THs, which vary for genetic reasons, drive the differences in both RMR and habitual PA.

THs regulate many metabolic processes, including cellular respiration, protein turnover, and energy metabolism (Yavuz et al., 2019). The chief forms of THs, T4 and T3 are secreted by the thyroid gland under the regulation of pituitary hormone TSH. In healthy humans, approximately 90% of the THs are released as the pro-hormone T4, and 10% is released as the active hormone T3. In target tissues, T4 is converted into its active form, T3, by the deiodinase enzymes DIO1 and DIO2 (Panicker, 2011; Fontenelle et al., 2016). The majority of the circulating THs (more than 99.7%) is attached to plasma proteins, including albumin, thyroxine-binding globulin (TBG), and thyroxine-binding prealbumin or transthyretin (TBPA) (Oppenheimer, 1968). A small percentage of circulating THs is found in free form: fT4 = 0.03% of total serum T4 and fT3 = 0.3% of total serum T3 (Mennitti et al., 2024). The T3 binds to specific thyroid hormone receptors in the nucleus or other thyroid receptors within the mitochondria to exert its diverse effects (Brent, 2012).

The influence of THs on energy expenditure components is well documented, particularly in overt thyroid conditions such as hyperthyroidism and hypothyroidism (Fox et al., 2008; Iwen et al., 2013). Hyperthyroidism is characterized by elevated serum T4 and T3 levels and is associated with an increased basal metabolic rate, often resulting in weight loss (Brent, 2008). Healthy underweight individuals (with BMI< 18.5) on average have greater than anticipated RMR, alongside higher levels of fT4 (Hu et al., 2022). In contrast, hypothyroidism reduces basal metabolic rate and total energy expenditure, leading to several metabolic disorders, including obesity (Meng et al., 2015). In addition to thyroid dysfunction, levels of THs within the euthyroid range are also associated with variations in resting metabolism (Astrup et al., 1996; Johnstone et al., 2005; Johannsen et al., 2012; Karkhaneh et al., 2019).

In the present study, we investigated cross-sectional correlations between RMR, habitual PA, and TH levels in a largely sedentary population of euthyroid females and their association with TEE. Specifically, we investigated whether the association between habitual physical activity and RMR is influenced by variability in TH levels.

## 2 Materials and methods

### 2.1 Participants characteristics

**Figure 1:**
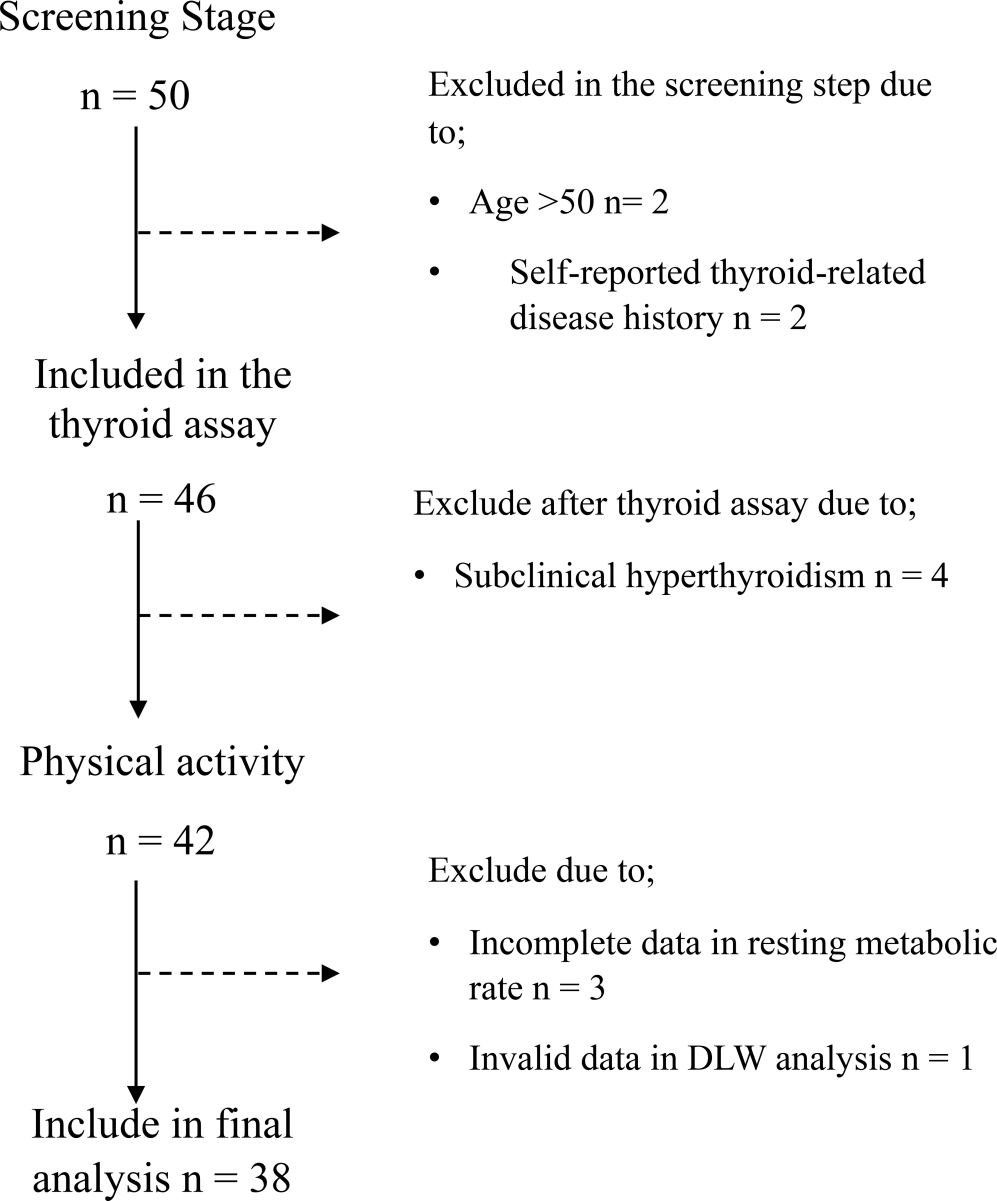
Flowchart of the participants included in the study. The dotted line represents the participant exclusion.

This cross-sectional study involved forty-two female participants average age of 30.31 (5.70), with a body mass index (BMI) ranging from 19.0 to 44.5 kg/m. Complete-case analysis included 38 participants with valid data for all primary variables to examine the relationship between PA, RMR, and TEE. The recruitment was done by advertising posters on community boards and on social media platforms (digital posters). Reflecting the local population, all recruited participants were Chinese. The data collection was done in the summer months (June-September), where the average ambient temperature was around 28 °C. Exclusion criteria included a history of thyroid disease (thyroid-related disorders determined using the health checkup reports provided by the participants at the screening stage), self-reported unintended weight changes of more than 2 kg in the past three months, pregnancy or breastfeeding, metal implants, and the use of special medical diets. During screening, several volunteers were deemed ineligible due to exceeding the age limit of 50 (n = 2), reporting a history of thyroid disease (n = 2), or having subclinical hyperthyroidism confirmed by thyroid assay (n = 4) (**Figure 1**). The Institutional Review Board of the Shenzhen Institute of Advanced Technology, Chinese Academy of Sciences (SIAT-IRB-240415-H0718) approved the study. Written informed consent was obtained from all participants.

### 2.2 Height, weight and body composition

All the participants visited the Centre for Energy Metabolism and Reproduction laboratory in the morning between 8 am and 9 am after an overnight fast (no less than a 10-hour fasting period). They were instructed not to consume caffeinated products before visiting the unit and to avoid heavy exercise (for at least 12 hours). The subject’s height (± 0.1) was measured in the standing position with bare feet using a stadiometer (Seca 217), and the weight (± 0.1) was measured using a digital scale.

The body composition, including the fat-free mass (FFM), fat mass (FM), and bone mineral content (BMC) was measured by dual-energy x-ray absorptiometry (DXA) (HOLOGIC Horizon Wi). Before performing the DXA, participants were provided with appropriate clothing and requested to remove all metal items they were wearing (e.g., jewellery, eyeglasses). The DXA measurements lasted for approximately 7 minutes, and quality control calibration was performed each morning before the measurements using a spine phantom.

### 2.3 Resting metabolic rate measurements

While participants were still in a fasting condition, their metabolic rate was measured using the indirect calorimeter with a ventilated hood system (COSMED Quark PFT ergo). Flow meter calibration and gas calibration were performed every morning before the test. Participants were instructed to keep still yet remain awake during the measurement period. Measurements lasted for 30 minutes while in a supine position in a quiet room (room temperature 24–26°C). Energy expenditure was calculated using the Weir equation (Weir, 1949) based on gaseous exchange rates of oxygen and carbon dioxide. The mean energy expenditure was calculated every 10 minutes, and the lowest value was taken as the subject’s metabolic rate (**Supplementary Figure 2A**). As reported in previous studies (Chen et al., 2020), sleeping metabolic rate (SMR) measured by the chamber calorimeter can serve as an alternative to RMR. Therefore, we were interested in the comparison of these two components of energy expenditure and measured SMR using a chamber calorimeter and RMR using a hood calorimeter. Initially, all participants were invited to complete both procedures. However, completion depended on participants’ availability because the chamber calorimeter required an overnight stay. Some participants were unable to stay overnight due to personal or scheduling constraints; therefore, they completed only the hood calorimetry. As a result, 39 participants completed hood calorimetry (RMR), 33 completed chamber calorimetry (SMR), and 30 completed both. However, to avoid the potential systematic bias, we only use the RMR measured from the hood calorimeter for further analysis. The metabolic chamber (OMNICAL, Maastricht instrument) is a sealed room with a continuous gas supply, providing 24-hour energy expenditure data. Each room is equipped with a bed, study desk, intercom, deep-freeze toilet, air conditioner, and a built-in sink. To ensure the volunteers’ safety, an alarm system was activated, and researchers monitored gas exchange throughout the experimental period. The room temperature was maintained at 25.0 ± 1.0. The oxygen and carbon dioxide measurements were converted into energy expenditure in kJ using the Weir formula (Weir, 1949). The total energy expenditure of the participants in MJ/day was plotted against time in 30-minute intervals over 24 hours (**Supplementary Figure 2B**). The lowest and most stable energy expenditure during sleep time was considered the RMR of the participants, which occurred from 2:00 am to 5:00 am.

### 2.4 Blood sampling and thyroid hormone levels

We did not specifically control for or collect samples in a particular phase of the menstrual cycle. Previous data only reported a subtle association with menstrual cycle function, particularly with sex steroid hormone levels in healthy women, and overall evidence for major thyroid fluctuations across the cycle is limited (Jacobson et al., 2018). However, according to some studies, T4 and T3 are comparable in the follicular phase and luteal phase of the menstrual cycle (Sharma et al., 2020). Fasted blood samples were collected by trained personnel from the brachial vein in the same morning after they finished the body composition measurements. The collected samples were kept at room temperature for 30 minutes to clot and then centrifuged at 2000 rpm at 4°C for 20 minutes to obtain serum. Aliquoted serum samples were then stored at -80°C for the batch analysis. TH levels were measured using a radioimmunological assay (Beijing North Institute of Biotechnology Co., Ltd). The reference range of each thyroid hormone was as follows: 0.9-2.2 ng/ml for T3, 45–135 ng/ml for T4, 3.18-9.22 fmol/ml for fT3, 8.56-25.60 fmol/ml for fT4 and 0.3-5.0 μIU/ml for TSH. The laboratory reference ranges of TSH and fT4 indicate normal thyroid function.

### 2.5 Physical activity and total energy expenditure

Physical activity was measured in free-living conditions. Participants were requested to wear a GT3x accelerometer (Actigraph Ltd, Pensacola, FL, USA) on their right hip 24 hours a day for 14 consecutive days, except during showering or swimming. After 14 days, participants returned their devices to the laboratory, and the data were downloaded using Actilife 6 data analysis software.

Participants who wore a GT3x for at least 4 days, more than 10 hours per day, were included in the analysis. Wear time was determined by subtracting the non-wear time from 24h. Non-wear time was filtered from the raw data based on zero vector magnitude counts (VM) per minute. VM per day was calculated after subtracting the non-wear time from the wear time. The PA measured by the device was analysed as the time spent in moderate to vigorous physical activity (MVPA), activity counts per day using triaxial VM counts, and daily physical activity intensity. Furthermore, the time spent on daily activities was categorised as light, moderate, vigorous, and very vigorous using the PA cut points. PA cut points were defined using the metabolic equivalent task (MET); 1-1.5 MET for sedentary, 1.5–3 MET for light PA, 3–6 MET for moderate PA and >6 MET for vigorous PA (Migueles et al., 2017).

Total energy expenditure was measured using the doubly labelled water method (DLW) (Speakman). Based on each subject’s weight, the dose of doubly labelled water (10% ^18^O and 5% ^2^H) was calculated to achieve an equilibrium of around 300ppm excess for ^18^O and 150ppm excess for ^2^H. Before the administration of the DLW dose, baseline urine samples were collected. Then, a pre-calculated dose was administered orally to each subject. The precise administration time was recorded, and three hours post-dose, the first urine sample was collected. Urine samples were collected for 14 days at as close to 24-hour intervals as possible. The precise collection time was recorded for all urine samples. The collected samples were stored at -20 °C until batch analysis. Before analysis, the urine samples were defrosted at room temperature and vortexed for 10 seconds. Approximately 150 μl of each urine sample was encapsulated into capillaries, which were then vacuum distilled. The water resulting from distillation was analysed using the liquid water analyser (ABB GLA431-TLWIA) (Melanson et al., 2018). Three international standards (Standard Light Artic Precipitate, Standard Mean Ocean Water, and Greenland Ice Sheet Precipitation) and four laboratory standards run alongside the samples for standardisation. The TEE was calculated using a recently derived equation that performed best in validation studies (Speakman et al., 2021). To account for the influence of body composition on the TEE, residual TEE values were generated using FFM and FM of the participants.

Physical activity level (PAL) and physical activity energy expenditure (PAEE) were calculated using the RMR and TEE of participants. The participants’ PAL was determined by the ratio of TEE and RMR (TEE/RMR) (Speakman and Westerterp, 2010). PAEE was calculated using the equation: PAEE = TEE - (0.1xTEE) - RMR, assuming the thermic effect of food is ~10% of the TEE (Schoeller and Jefford, 2002; Speakman et al., 2021).

### 2.6 Statistical analysis

Statistical analysis was performed using GraphPad Prism 8.0.1 and R Studio (version 4.3.3). Continuous variables are expressed with mean and SD. Model assumptions were verified through examination of residual normality using Shapiro-Wilk tests, Q-Q plots, a homoscedasticity test using the Breusch-Pagan test (BP), independence assessment check via Durbin-Watson and multicollinearity with the variance inflation factors (VIF).

Correlation analysis between TH parameters was performed using Spearman's correlation coefficients. The model examining the determinants of thyroid hormone levels included body composition (FFM, FM), age and BMC as covariates. Other models include FFM and FM as covariates.

To determine the associations between thyroid hormones and RMR and TEE we employed a comprehensive regression modelling strategy. First, we conducted a bidirectional stepwise regression using the “orlss” package in R with RMR and TEE as outcome variables. The entry p-value was set at 0.05, and the removal p-value was set at 0.1. Then we conducted a set of theory-driven models to analyse the robustness of the stepwise models. Those models include following covariates: Model 1: body composition and age (FFM, FM, and age), Model 2: body composition and thyroid hormones (FFM, FM, T3, T4), Model 3: body composition and PA (FM, FM, PA), and Model 4: a full model (FFM, FM, T3, PA). Equivalent models were also applied with TEE as the outcome; Model 1_TEE: body composition and age (FFM, FM, and age), Model 2_TEE: body composition and RMR (FFM, FM, RMR), Model 3_TEE: body composition and PA (FM, FM, PA), Model 4_TEE: body composition and THs (FFM, FM, T3, T4). Model 5_TEE: a full model (FFM, FM, RMR, PA, T3, T4). The theory-driven and the final stepwise models were assessed using the Akaike Information Criterion (AIC) values.

To examine the statistical associations between PA, THs, and RMR, we used “lavaan” package in R. We tested two competing directional models using structural equation modelling (SEM). The hypothesised model examined physical activity → thyroid hormones → metabolic rate, while the reverse model tested metabolic rate → thyroid hormones → physical activity. Both models used a thyroid hormone composite score and were estimated with maximum likelihood with 5000 bootstrap samples. SEM analyses rely on both statistical assumptions (linearity, normally distributed residuals, homoscedasticity, independence, and absence of problematic multicollinearity or influential outliers) and conceptual assumptions such as temporal ordering of variables and no unassumed confounding. Statistical significance was set at p< 0.05 for all analyses.

## 3 Results

### 3.1 Thyroid hormone

The final analysis included 38 participants’ thyroid hormone data. The correlations between thyroid hormones are illustrated in **Supplementary Figure 4**, and clinical characteristics of participants are shown in **Table 1**. Since THs are highly correlated, we performed a correlation analysis, and a strong positive correlation was observed between total T4 and fT4 (r = 0.87) (**Supplementary Figure 4**). We analysed the association between each thyroid hormone parameter and FFM, FM, age and BMC as predictors using multiple regression analyses. fT3 was positively associated with FM (p = 0.041), TSH was positively associated with FFM (p = 0.021), and TSH was significantly negatively associated with BMC (p = 0.04). However, other THs had no significant association with these factors.

**Table 1:** Subject characteristics.

Characteristics	Summary n = 42 mean (SD)	Final analysis summary n = 38 Mean (SD)
Age (Y)	30 (5.70)	31 (5.82)
Height (cm)	161.0 (7.31)	160.6 (7.60)
BMI (kg/m^2^)	27.63 (5.79)	27.83 (5.50)
Fat mass (kg)	32.06 (10.12)	32.05 (9.80)
Fat-free mass (kg)	39.27 (7.89)	39.87 (7.53)
Bone mineral content (g)	2050 (329.7)	2059 (333.7)
Physical activity (log_e_ vector magnitude counts per day)	12.94 (0.37)	12.84 (0.36)
Thyroid parameter		
T3 (ng/ml)	1.30 (0.37)	1.23 (0.25)
T4 (ng/ml)	88.41 (18.97)	87.37 (18.88)
fT3 (fmol/ml)	6.19 (1.40)	6.06 (1.25)
fT4 (fmol/ml)	15.23 (2.85)	15.00 (2.88)
TSH (μIU/ml)	1.09 (0.87)	1.05 (0.78)
Components of energy expenditure		
TEE (MJ/day)	–	9.14 (2.0)
PAEE (MJ/day)	–	1.98 (1.2)
PAL	–	1.46 (0.2)
RMR (MJ/day) Hood Calorimeter	–	6.23 (0.99)

Values are presented as mean (SD). SD, standard deviation; BMI, body mass index; T3, triiodothyronine; T4, thyroxine; fT3, free triiodothyronine; fT4, free thyroxine; TSH, thyroid-stimulating hormone; TEE, total energy expenditure; PAEE, physical activity energy expenditure; PAL, physical activity level; RMR, resting metabolic rate.

### 3.2 Resting metabolic rate

The mean RMR of the participants measured from the ventilated hood system was 6.2 MJ/day (SD = 0.99, CV = 15.8%, n = 38). The maximum and minimum values were 9.6 MJ/day and 4.7 MJ/day, respectively. For the participants who were measured in the metabolic chamber (n = 33), the mean was 6.2 MJ/day (SD = 1.0, CV = 16.6%), with minimum and maximum values of 4.5 MJ/day and 9.3 MJ/day, respectively. Thirty individuals underwent both the hood and chamber method measurements. These data were used to compare the two approaches using Bland-Altman and reduced major axis plots (**Supplementary Figures 3A, B**). The Bland-Altman analysis revealed that the Hood method slightly overestimated RMR relative to the chamber method (mean = 0.22). Despite this, the methods were strongly correlated (r² = 0.66, p< 0.0001). Since the majority of the participants completed the hood method measurements, that data was used for further analysis.

FFM and FM accounted for most of the individual variation in RMR. FFM alone explained 69.0% of the variation of RMR (p< 0.001) (**Supplementary Figures 5A–C**). A multiple linear regression model including FFM (β = 0.61) and FM (β = 0.25) explained 70.8% (**Supplementary Figure 6A**) of the variation of the RMR. Age was not statistically significantly associated with the resting metabolic rate (p = 0.49, β = -0.06).

**Figure 2:**
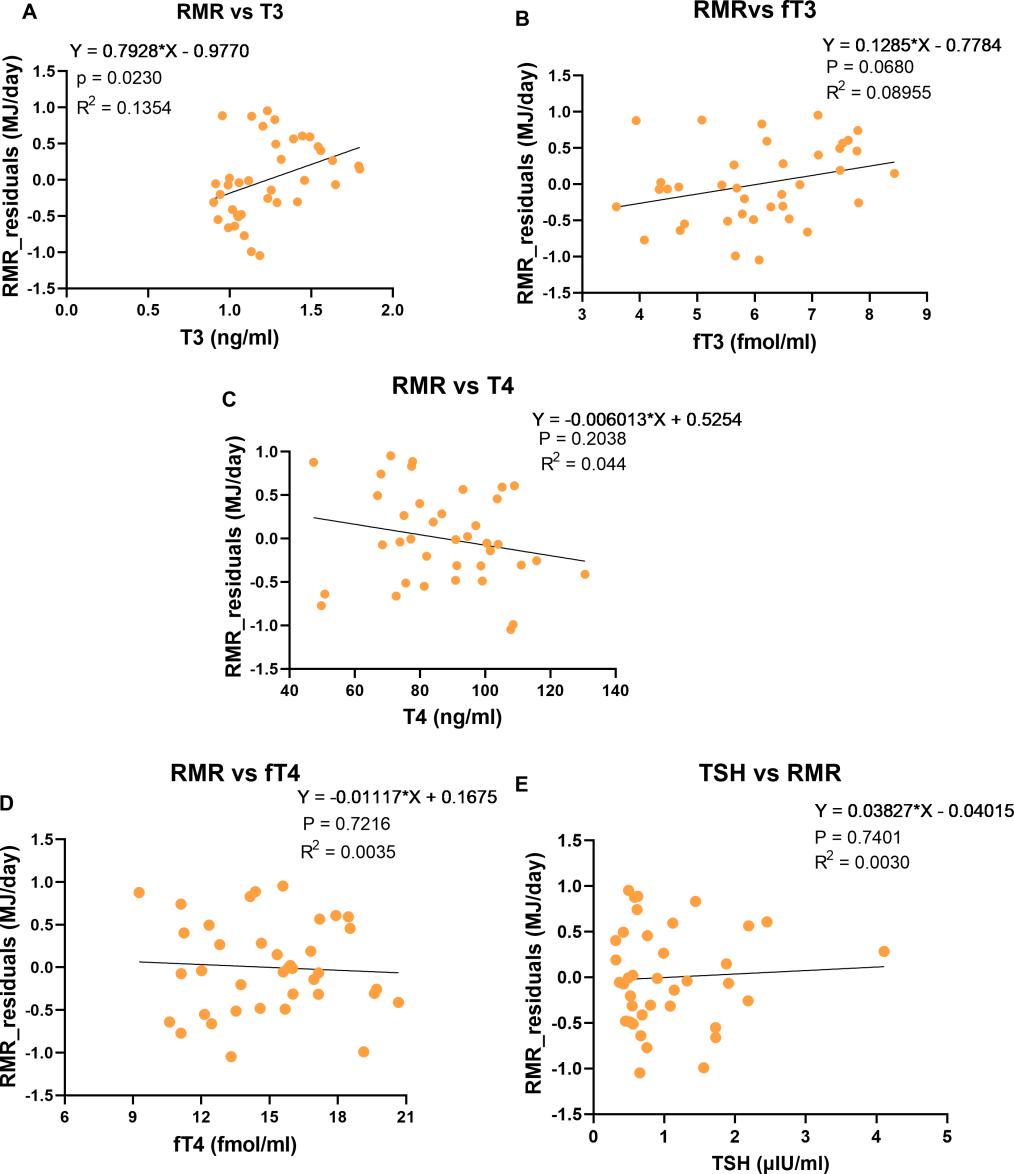
Association between Thyroid hormone and the residual RMR (resting metabolic rate adjusted for body composition). **(A)** Association between T3 and residual RMR, **(B)** Association between fT3 and residual RMR, **(C)** Association between T4 and residual RMR, **(D)** Association between fT4 and residual RMR, **(E)** Association between TSH and residual RMR. Only participants measured with hood calorimetry were included (n = 38).

We removed the effect of FFM and FM on the RMR by generating residual RMR values. Then, we sought the association between the residual variation of RMR and each thyroid hormone. The residual RMR was positively associated with T3 (p = 0.02, β =0.37) and approached significance with fT3 (p = 0.07, β = 0.29). Other thyroid parameters were not significantly related to residual RMR in univariate analysis (**Figures 2A–E**). Stepwise regression was conducted with all TH together, revealing that T3 had a significant positive association (p = 0.02) and explained 13.5% of the residual variation of RMR. The model was evaluated for statistical assumptions (**Supplementary Table 2**; **Supplementary Figures 6D–F**).

### 3.3 Physical activity

The final analysis of physical activity included 38 volunteers’ accelerometer data. PA data were log_e_-transformed before the analysis. The log_e_ converted activity counts per day (log_e_ vector magnitude counts -log_e_ counts) had a mean value of 12.84 log_e_ counts (SD = 0.4, CV = 2.8%) with a maximum of 13.8 and a minimum of 11.6 log_e_ counts. Hourly VM and MVPA differed significantly across the day, though both followed a similar daily pattern (**Supplementary Figures 8A, B**). Analysis of daily activities revealed that this group of participants spent around 95.5% of their time engaging in light physical activities and approximately 4.2% moderate physical activities, while vigorous and very vigorous activities together accounted for less than 1% of overall physical activity.

**Figure 3:**
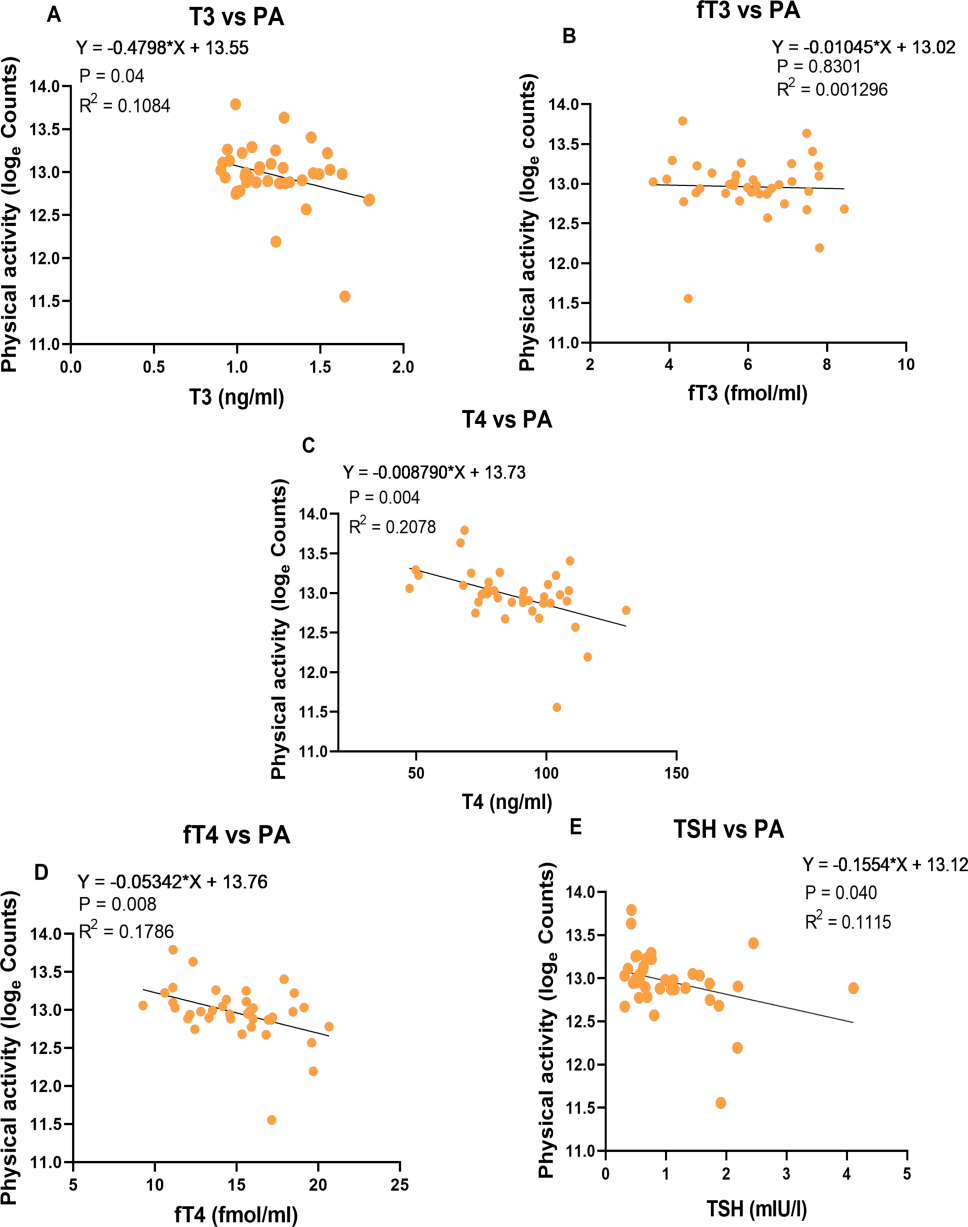
Association between Thyroid hormone and physical activity. **(A)** Association between T3 and PA, **(B)** Association between fT3 and PA, **(C)** Association between T4 and PA, **(D)** Association between fT4 and PA, **(E)** Association between TSH and PA. Physical activity (loge count/d); vector magnitude loge counts per day (n = 38).

Multiple regression models of PA with FFM, FM and age as factors revealed no significant associations between physical activity and those morphological traits. In univariate linear regression models, PA was negatively associated with T3, T4, fT4, and TSH, but not significantly associated with fT3. (**Figures 3A–E**). When the THs were entered into stepwise regression, circulating thyroid hormone levels explained 20.8% of the variation in physical activity, and the model retained a significant negative association with T4 (p = 0.004, β = -0.46) (**Supplementary Figure 6G**).

SEM was employed to test statistical relationships between PA, RMR, and THs. In the hypothesised direction ( PA→TH→RMR), PA showed a significant negative association with THs levels (β = -4.335, 95% CI [-1.70, -8.51], p = 0.011). The pathway from THs to RMR was positive but non-significant (β = 0.043, 95% CI [-0.02, 0.10], p = 0.16). The indirect effect was non-significant (β = -0.186, 95% CI [-0.67, 0.09], p = 0.31). In the reverse model ( RMR→TH→PA), RMR showed a significant direct effect on PA (β = 0.174, 95% CI [0.04, 0.38], p = 0.046), while the pathway from RMR to THs was non-significant (β = 0.840, 95% CI[-1.23,3.32], p = 0.46). The indirect effect in this direction was non-significant (β = -0.043, 95% CI [-0.23, 0.06], p = 0.55). The direct effect of PA on RMR was marginally significant (β = 0.472, 95% CI [-0.11,1.18], p = 0.083). These results highlight the interrelated nature of PA, RMR, and THs (**Table 2**).

**Table 2:** Results of SEM analysis.

Path	Estimate	Standard error	z-value	p-value	95% Confidence interval	Std. estimate
SEM 1 ( PA→TH→RMR)						
Direct effect (c) PA→RMR	0.47	0.27	1.73	0.08	[0.11, 1.18]	0.319
PA→TH (a)	-4.33	1.71	-2.53	0.01	[-8.513, -1.703]	-0.456
TH→RMR (b)	0.04	0.03	1.41	0.16	[-0.02, 0.10]	0.28
Indirect effects (a*b) ( PA→TH→RMR)	-0.19	0.18	-1.005	0.31	[-0.67, 0.09]	-0.13
Total effects	0.29	0.22	1.29	0.20	[0.01,0.91]	0.19
SEM 2 (reverse) (RMR →TH→PA)						
Direct effect (c)	0.17	0.09	1.99	0.05	[0.04, 0.38]	0.26
TH→RMR (a)	0.84	1.14	0.74	0.46	[-1.2, 3.32]	0.13
TH→PA (b)	-0.05	0.02	-2.74	0.006	[-0.09,-0.09]	-0.49
Indirect effect (a*b) (RMR→TH→PA)	-0.043	0.07	-0.60	0.55	[-0.23, -0.06]	-0.06
Total effects	0.13	0.07	1.84	0.06	[0.002,0.28]	0.19

Results are presented for two structural equation models assessing bidirectional associations between physical activity (PA), thyroid hormones (TH), and resting metabolic rate (RMR). Standard errors and confidence intervals were estimated using 5,000 bootstrap samples. Direct, indirect, and total effects are reported alongside standardized estimates. TH, thyroid hormone composite score; PA, log-transformed vector magnitude; RMR, residual RMR adjusted for body composition.

The mean value of measured TEE using DLW was 9.1 MJ/day (SD = 2, CV = 22.21%, n = 38). The lowest TEE value was 5.8 MJ/day, and the highest was 15.1 MJ/day. The TEE measured in the chamber had a mean of 7.6 MJ/day (SD = 1.2, CV = 16.50%, n = 33). The maximum and minimum values were 11.3 MJ/day and 5.7 MJ/day, respectively. Both TEE from DLW and the chamber were significantly positively associated with FFM (p< 0.0001) and FM (p< 0.0001), indicating that, similar to RMR, inter-individual differences in TEE are largely explained by variations in body composition (**Supplementary Figures 9A–C**). We obtained completed data for both DLW and Chamber TEE for twenty-nine individuals. These data were used to compare the two approaches using the Bland-Altman and reduced major axis plots (**Supplementary Figures 3C, D**). The Bland-Altman analysis revealed that the DLW method gave a higher estimate of TEE than the chamber, with a mean difference of 1.69 MJ/day. Reduced major axis regression confirmed a strong linear agreement between the two methods with r^2^ = 0.61 and p< 0.0001. Since DLW is considered more reflective of habitual expenditure in daily life, it was used for further analysis.

A multiple linear regression model including FFM (β = 0.80) and FM (β = 0.02) explained 66.5% (**Supplementary Figure S11A**) of the variation in TEE, while age was not significant (p = 0.27, β = 0.11). The residual variation of TEE plotted against each TH; however, none of the THs were significantly associated with residual TEE (**Supplementary Figure 10**).

**Figure 4:**
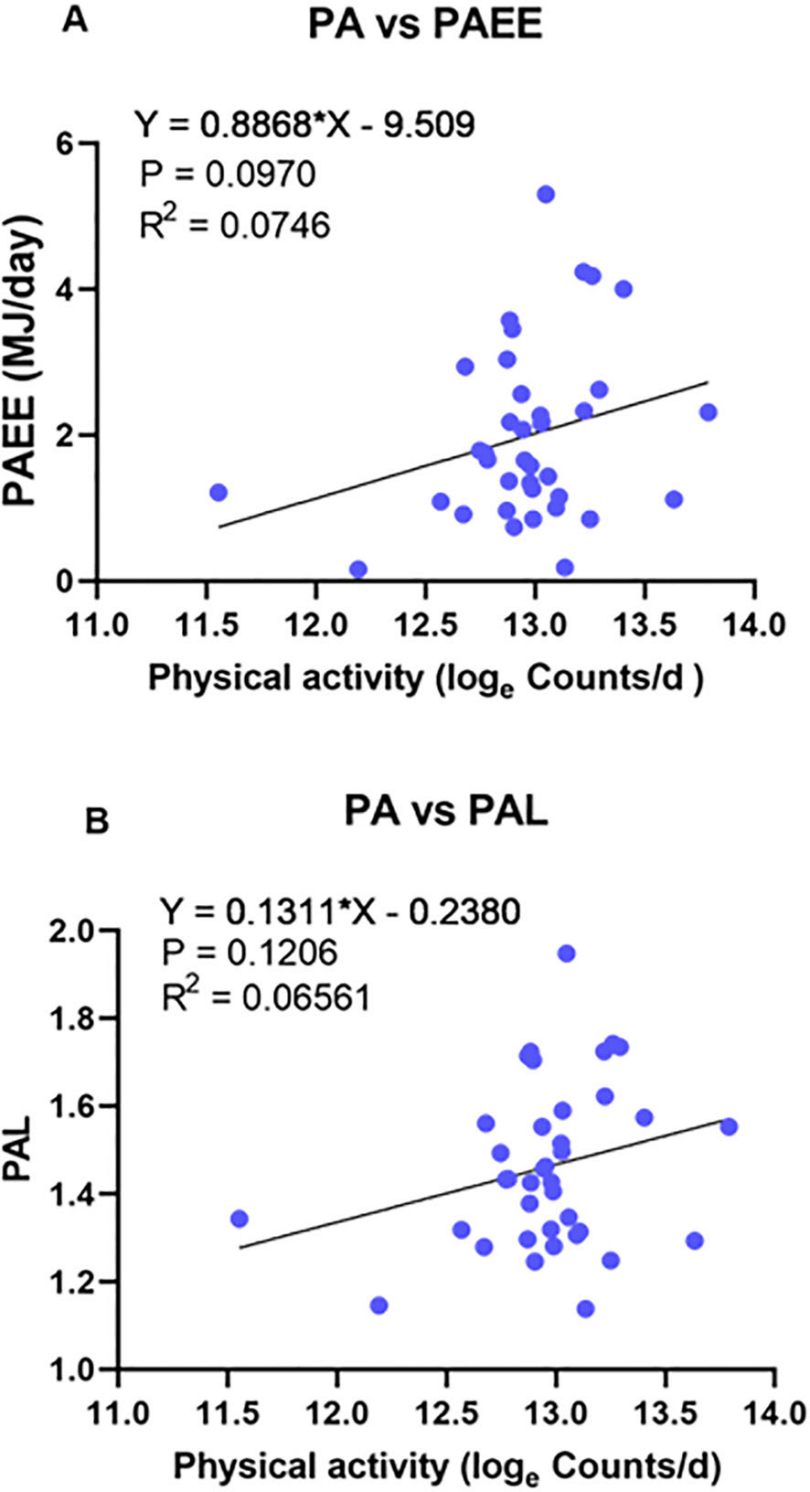
Relationships between physical activity assessed by doubly labelled water (DLW) and accelerometry-derived activity counts. **(A)** Physical activity energy expenditure (PAEE) vs. accelerometer-based physical activity (PA). **(B)** Physical activity level (PAL) vs. accelerometer-based PA. PAEE and PAL were calculated using DLW data, whereas PA was estimated from vector magnitude counts derived from accelerometer recordings.

The calculated PAEE (0.9*TEE-RMR) had a mean of 1.98MJ/day (SD = 1.2, CV = 59.51%) with a minimum of 0.16 MJ/day and a maximum of 5.3 MJ/day. In the univariate linear regression model including PAEE as the outcome and PA as predictors, the association with PA was not significant (p = 0.097) (**Figure 4A**). The mean PAL was 1.46 (SD = 0.2, CV = 12.75%), ranging from 1.14 to 1.95. The association between PAL and PA was also not statistically significant (p = 0.12) (**Figure 4B**). Both PAEE (p = 0.0003) and PAL (p =0.04) were significantly associated with FFM, while FM showed a significant association with PAEE (p = 0.005) (**Supplementary Figures 13A–D**).

**Figure 5:**
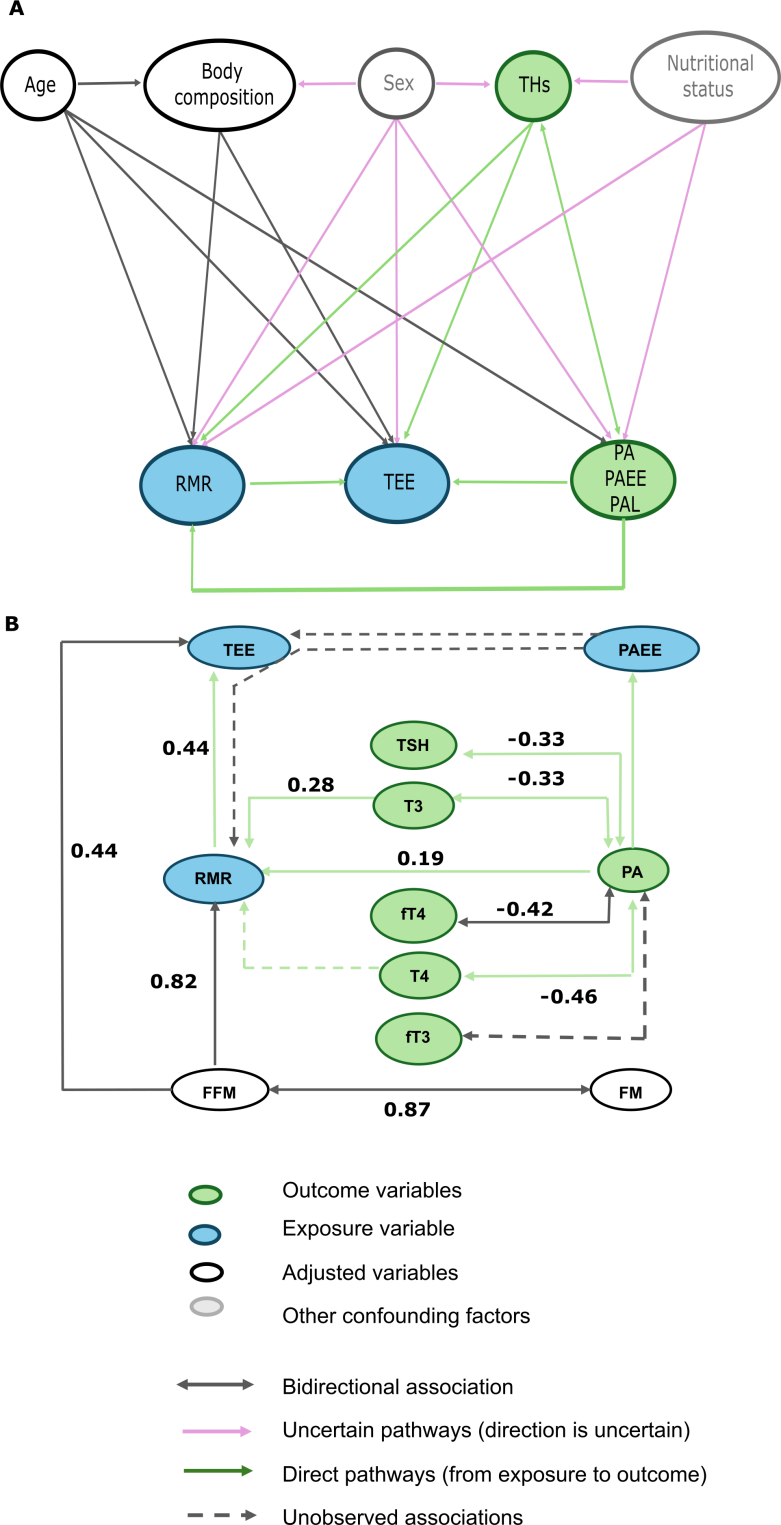
Relationship between the energy expenditure traits (RMR, PAEE, PAL and TEE) PA, circulating TH concentrations and body composition. **(A)** Directed Acyclic Graph indicating possible confounders of the energy expenditure and thyroid hormone. In the current study, nutritional status and sex differences were not measured, as they might influence the individual variation of TH and energy expenditure; they are included in the diagram. Arrows represented a statistical association between the variables rather than a causal relationship. Green arrows indicate a direct pathway between the exposure and outcome variables. Pink arrows indicate uncertain directions (among the variables that are not included in the study and the outcome and exposure variables), while the black arrows show the association between adjusted variables and exposure and outcome. **(B)** Simplified diagram. Possible yet unobserved pathways are presented in dashed lines. Arrows represent a statistical association rather than a causal relationship. Arrows are annotated with the observed correlation coefficients.


Finally, we sought the associations between the energy expenditure traits (RMR, PAEE, PAL, and TEE), PA, and the circulating TH concentrations, while accounting for the body composition (**Figure 5**). Alongside FFM (p<0.0001), which remained the strongest predictor of RMR, there were significant positive effects of T3 (p = 0.002) and PA (p = 0.04) on RMR. No other thyroid markers (T4, fT3, fT4, TSH) or FM significantly contributed to the variation of resting metabolism. The model suggested that 77.4% variation in RMR could be explained by body composition, PA, and circulating TH. Thus, the variation of RMR in our population was best described as;

$$\text{RMR }MJday^{-1}=\;-6.23\;+\;0.52\text{PA }log_{e}counts/d\;+\;1.14\text{T}3\;ngml^{-1}\;+\;0.11\text{FFM }kg$$

$$({\text{r}}^{2}=\;0.774,\text{ p }<0.0001).$$

A set of theory-driven models was generated to analyse the robustness of the stepwise model (**Supplementary Table 3**). Across all theory-driven models, FFM, consistently showed a positive association with RMR. In Model 2, T3 was a significant predictor of RMR (β = 0.24, p = 0.0095), while T4 approached significance (β = -0.18, p = 0.06). In model 3, PA was not significant with RMR (β = 0.11, p = 0.25). In model 4, both T3 (β = 0.27, p = 0.004) and PA (β = 0.19, p = 0.035) remained significant independent predictors, indicating that their associations with RMR were not dependent on model structure. Overall, FFM, T3, and PA emerged as consistent predictors of RMR across both theory-driven models and stepwise regression. The comparison of the theory-driven model and the final regression model was done using AIC values. According to the results final stepwise model has the lowest AIC value (AIC = 59.6) (**Supplementary Figure 7**).

When using RMR values obtained from the metabolic chamber, only FFM (p<0.0001) emerged as a significant predictor, while THs and PA did not significantly contribute.

A multiple regression model including THs, FFM, FM and PA as predictors of PAEE approached significance with PA (p = 0.08). The model suggested that a 44.3% variation in PAEE could be explained by circulating THs, physical activities, and body composition. A similar trend was observed with PAL and PA (p = 0.09) after including THs.

A stepwise regression approach was employed to identify the significant predictors of TEE from RMR, PA, THs and body composition. The model retained RMR, and FFM, and it explained approximately 70.7% of the variance in TEE, with significant positive associations for RMR (p = 0.01), FFM (p = 0.01) (**Supplementary Figures 11D–F**). Model diagnostic presented in **Supplementary Table 4**. The associations could be presented as follows;


$$\text{TEE }MJday^{-1}=\;-1.25\;+\;0.91\text{ RMR }MJday^{-1}+\;0.12\text{ FFM kg }\left({\text{ r}}^{2}\;=\;0.71,\text{ p}<0.001\right)$$

A set of theory-driven models was generated to analyse the robustness of the stepwise model (**Supplementary Table 5**). Across all theory-driven models, FFM, consistently showed a positive association with TEE. In Model 4_TEE, T3 was a significant predictor of TEE (β = 0.21, p = 0.044), together with FFM (β = 0.81, p = 0.0003). In Model 2_TEE, RMR was significant with TEE (β = 0.45, p = 0.011). In model 5_TEE,only FFM was significant with TEE (β = 0.63, p = 0.009). Overall, FFM emerged as a consistent predictor of TEE across both theory-driven models and stepwise regression. The comparison of the theory-driven model and the final regression model was done using AIC values. According to the results final stepwise model has the lowest AIC value (AIC = 121.3) (**Supplementary Figure 12**).

## 4 Discussion

There are two main indirect calorimetry methods for measuring resting metabolism in humans, among which hood calorimetry is the most commonly employed. We used both hood calorimetry and chamber calorimetry to evaluate RMR. It was suggested that SMR could be used as an alternative to RMR (Chen et al., 2020). In our study, we assessed the agreement between these two methods using Bland-Altman analysis. Consistent with previous findings, SMR measured via the chamber calorimeter was closely related to the RMR values derived from the hood calorimeter (Speakman and Selman, 2003; Wouters-Adriaens and Westerterp, 2006).

There is no clear definition for SMR. However, some have proposed SMR as the average of the lowest and most stable energy expenditure detected over three consecutive hours, while others have defined it as the average minimal energy expenditure over a predetermined post-prandial period, such as 0100–0500 h or 0200–0500 h (Wouters-Adriaens and Westerterp, 2006; Chen et al., 2020). Following previous studies, we defined SMR as the average energy expenditure between 0200–0500 h. These SMR values were closely related to the RMR measured via the hood method, as further clarified by the Bland-Altman analysis, with a mean of 0.22 and a limit of agreement between -0.97 and 1.47.

The most metabolically active component of the body, FFM, was a robust predictor of RMR and TEE (Carneiro et al., 2016; Pontzer et al., 2021). However, the role of FM in the variability of the components of energy expenditure remains debated, with some studies finding a significant independent effect on RMR (Johnstone et al., 2005) and others failing to reveal such an association (Karkhaneh et al., 2019). Our work suggested that, in addition to FFM, there was also a small independent effect of FM on RMR. In multiple regression, the RMR coefficient for FFM was 0.11, almost twice that of FM at 0.07, indicating FFM’s greater impact on RMR. Nevertheless, due to high collinearity between the FFM and FM, isolating the true contribution of either factor remains challenging. Age is another commonly observed predictor of the RMR and TEE. Previous studies have proposed that age is inversely related to the RMR (Fukagawa et al., 1990; McMullan et al., 2016). However, others reported that total and basal metabolism, including FFM, were stable in adults (20–60 years) (Pontzer et al., 2021). In agreement with those latter findings, we did not observe a significant difference in the RMR or TEE with the age (22–42 years) of this group of adults.

The production of THs is regulated by the negative feedback loop of the hypothalamic pituitary axis (HPT axis), where circulating THs (T3) suppress TSH secretion to maintain the hormonal balance in euthyroid individuals (Hall et al., 1970; Morley, 1979). The association between variation in serum thyroid levels and metabolic rate differences has long been established. THs fine-tune metabolic pathways such as gluconeogenesis, lipogenesis, and thermogenesis, which in turn influence the RMR (McMullan et al., 2016). However, previous work suggests this relationship exhibits sexual dimorphism. For example, Johnston et al. (2005) reported a significant relationship between T4 and relative metabolic rate in men but not in women (Astrup et al., 1996; Johnstone et al., 2005). Another study in males with eating disorders has shown that reduced energy availability is accompanied by lower T4 levels (Skolnick et al., 2016). In contrast, a study on females reported a significant association with RMR and T3. Consistent with these studies, we observed a significant association between RMR and T3 (p = 0.02), but not with T4, in our sample of females. Taken together, these findings suggest that T3 may be a more physiologically relevant biomarker for assessing metabolic function in females than T4. Mechanistically, this sex-specific pattern is plausible. T3 is the biologically active form of thyroid hormone, while T4 serves as a prohormone. A healthy adult needs 30µg of T3 daily, and 25µg of it is produced in the peripheral tissues by DIO enzymes (Bianco et al., 2002). Among the DIO enzymes, DIO2 is believed to produce most of the T3 (Luongo et al., 2019). Previous animal studies have reported a sex difference in DIO enzyme activity, with an observed trend of DIO2 stimulated by estrogen (Lisbôa et al., 2001), and higher DIO2 expression was reported in the female mice’s brain (Baksi and Pradhan, 2021). Moreover, T3 actively influences metabolism by regulating mitochondrial activity and adaptive thermogenesis (Sagliocchi et al; Drigo et al., 2013). Sex hormones further contributed to the higher observed thyroid disorders in females. It is argued that higher autoimmune thyroid diseases may be related to estrogen. Reviewed by Xie et.al, estrogen and its receptors are involved in enhancing the thyroxin-binding globulin (TBG) production, and stimulating the HPT axis to increase TSH secretion (Xie et al., 2025). However, a recent study on thyroid hormone across the menstrual cycle observed that serum T3 and T4 concentrations were comparable in the follicular and luteal phases in the menstrual cycle (Sharma et al., 2020). Although we did not specifically assess menstrual cycle effects in our cohort, these findings highlight that thyroid–hormone regulation might be sex-specific. In addition, inter-individual variation of TH depends on several other factors, such as age, body composition, and activity levels. A cross-sectional study on euthyroid men and women found that the fT3 and fT3 to fT4 ratio (fT3/fT4) were positively associated with BMI (Roef et al., 2014). Similarly, many previous studies have demonstrated changes in TSH (Song et al., 2019; Dai et al., 2020) and fT3 (De Pergola et al., 2007; Taylor et al., 2016) with BMI. However, it is not clear whether the changes in thyroid hormone in euthyroid status preceded the weight changes or the impact of fatness on the pituitary-thyroid axis drives the association. In our study, we did not assess the relationship between TH and BMI because BMI is a poor measure of body composition. Instead, we looked into the association between body composition and THs using more direct and accurate composition measures. We found a positive association between fT3 and FM and TSH with FFM but not with other thyroid parameters. Contradictory conclusions were reported in some previous studies. For example, Adamska et al. (2022) reported a negative connection between TSH and visceral adipose tissue (VAT) in lean men, while serum concentration of fT3 was positively associated with android and gynoid fat mass in overweight/obese men (Adamska et al., 2022). They did not find any association between FM and THs in women. In agreement with our results, a large cohort study on hypothyroid patients undergoing treatments with levothyroxine (LT4) also reported an association between fT3 and FM (Samuels et al., 2017).

In addition, THs are crucial in linear growth and skeletal development. We identified a relationship between bone mineral content and TSH. These results are consistent with the previous studies (Nappi et al., 2022), indicating that THs play a vital role in linear growth and skeletal development.

Thyroid receptors are essential regulators of skeletal muscle genes, and they are associated with muscle dysfunction in both humans and rodents (Nappi et al., 2022). A recent study on mice indicated that THs regulate the lipid content of the muscles and modify the ratio of saturated and unsaturated fatty acids in skeletal muscles’ lipid membranes, which impacts physical performance (Miro et al., 2023). To our knowledge, only a few studies have assessed the interaction between thyroid hormones and habitual PA. Most studies were interested in the exercise intervention and THs function, but did not assess the daily PA (Opstad et al., 1984). However, the majority of those studies that assess habitual PA rely on self-report to evaluate PA by a questionnaire (Roa Dueñas et al., 2021; Lee et al., 2024). In contrast, in our study, we utilised triaxial data provided by the accelerometer to assess PA, which provides more accurate data by capturing the movements across three planes. The results indicate significant negative associations between T4, T3, fT4 TSH and physical activity counts. These results are consistent with the previous studies (Ciloglu et al., 2005; Tian et al., 2024). TH levels can also affect daily activities. This reflects in hyperactivity in hyperthyroid people and lower activities in hypothyroid individuals (Taylor et al., 2018). Furthermore, the association between genetically determined variations in thyroid function and clinical outcomes highlights the importance of thyroid activity in metabolism (Sterenborg et al., 2024). A study on skeletal muscle physiology indicated TH can influence the fatty acid composition of skeletal muscle, thereby affecting exercise performance (Miro et al., 2023). Similarly, muscle contractile and metabolic properties are likely to be induced by DIO enzymes (Dulloo, 2025). Altogether, these evidences suggest a bidirectional role of THs and activities.

Thyroid hormones are key players in energy expenditure. Especially in thyroid disorders, it reflects altered metabolic functions and energy balance (Iwen et al., 2013). We assessed the daily energy expenditure via DLW and the gaseous exchange in chamber calorimeters. Respiratory chambers have been used as a tool to validate the measurements from the DLW method (Schutz, 2018). As anticipated, because DLW measures of TEE include much greater levels of physical activity and exposure to more diverse climatic conditions than in the chamber, the TEE by DLW was 1.69 MJ/day higher, approximately 18.6%.

We explored the association between RMR and PA, THs, and body composition, using both stepwise regression models and the theory-driven models. According to the models, FFM, T3 and PA showed a positive association with RMR, indicating that women with elevated habitual activity and higher circulating T3 levels tended to have a greater resting metabolic rate. However, to test a potential statistical association (pathway), specifically, whether thyroid hormones associate with the link between PA and RMR, we conducted an SEM analysis. This analysis did not support the proposed ( PA→TH→RMR)association. The indirect effect of PA on RMR through thyroid hormones was not statistically significant (indirect effect: β = -0.186, p = 0.31). This is further corroborated by the reverse model, which also showed a non-significant indirect effect from RMR to PA through THs (p = 0.55). Notably, we observed a consistent and significant negative association between PA and THs in both analytical directions. However, as this is a cross-sectional study, we cannot identify the direction of causality. Therefore, while PA and THs are robustly related, THs play only a limited role in the association of PA with RMR.

In the TEE regression model, which included RMR, PA, THs, and body composition as factors, RMR and FFM were retained as significant positive predictors, while THs and PA did not contribute significantly. The observed negative association between PA and THs could be explained by several mechanisms. First, there might be a compensatory mechanism where higher thyroid function might reduce voluntary activity. Second, as suggested by the constrained model of energy expenditure, higher levels of PA might reduce metabolic activity to maintain the TEE in a narrow range (Pontzer, 2015; Pontzer et al., 2016; Pontzer, 2017; Pontzer, 2018). The constraint of energy expenditure was first proposed after observing the similar adjusted TEE between the highly active Hadza population and the Western population (Pontzer et al., 2012). Similar results were observed in athletes in endurance events, where the measured TEE (using DLW) was lower than the predicted TEE (Thurber et al., 2019). Suggesting that increased activity levels potentially reduce the other components of TEE (Thurber et al., 2019). This would involve downregulating the non-essential expenditures, including suppression of the thyroid axis. Although we observed a significant negative relationship between TH levels and PA (p = 0.011), a corresponding decline in RMR was not detected. Furthermore, the non-significant indirect effect of THs (p = 0.31) suggests THs play only a limited role in the association between PA and RMR. The stability of RMR with PA is the default assumption of the classic additive model (Pontzer et al., 2012; Pontzer, 2015). A recent observational study further supported this additive relationship (Howard et al., 2025). Further, we did not find a significant association between either PA and PAEE or PA and PAL after accounting for the body composition in multivariate analysis. Previous studies have also indicated very weak associations between PAEE and objectively measured PA (Hu et al., 2022). The complexity in interpreting what PAEE and PAL actually represent has also been previously highlighted (Dai et al., 2020). However, interestingly, after adding THs, PA showed a trend toward significance with PAEE (p = 0.07), and PAL (p = 0.08). This suggests calling these PAEE and PAL, which emphasises that the effect of PA might be a misnomer. Perhaps PAEE would be better called non-resting energy expenditure (NREE) and PAL called metabolic scope, as is often used in studies of animals (Gavini et al., 2014). Nevertheless, we did not observe that the TEE reached a plateau with increased PA. According to data from large DLW study, a non-linear association between TEE and PA was observed after a proposed threshold of ~230 activity counts per minute per day (CPM/d). Before the cut point, the TEE and PA varied linearly (Pontzer et al., 2016). Our sample of adults was in the lower PA range (1.0–3 MET), reflects sedentary to light activity patterns and lower than the proposed threshold. Therefore, we did not observe a constraint. A recent large-scale study on human energy expenditure across the lifespan saw a quite stable activity pattern (moderate and vigorous), PAEE and PAL through adulthood (20–60 years) (Pontzer et al., 2021). In agreement with those results, we did not observe an age-related variation in either PAEE or PAL. There are several limitations to this study. First, the cross-sectional design of the study makes it difficult to conclude the temporal order among variables making it impossible to determine the causal relationship between PA and RMR via THs. Therefore, longitudinal studies are needed to test the direction of causality. Second, the study population was restricted to females; therefore, this may impact the generalizability of the findings, as previous work suggests that females and males may exhibit different patterns of association between thyroid hormone levels, energy expenditure, and physical activity levels. Additionally, thyroid hormones can vary across the menstrual cycle, and we did not standardize measurements to a specific phase. Future studies should control for menstrual cycle phase to minimise hormonal influence. The third limitation is related to the physical activity measurements. PA was measured using the GT3 device without an accompanying activity log, which prevented tracking activities such as swimming when it was necessary to remove the logger to prevent damage. As a result, total physical activity may in theory have been underestimated, but later follow-up with the participants indicated none engaged in swimming during the study. In addition, there are considerations regarding sample size and SEM. We calculated the sample prior to the study using power analysis to achieve sufficient power (see **Supplementary Document** for details). However, SEM, particularly when testing indirect effects, can benefit from larger samples to ensure stable parameter estimation and robust model fit (Abu-Bader and Jones, 2025). Although the present model was theory-driven and limited in complexity, our findings should be replicated in larger samples to strengthen confidence in the observed relationships. Lastly, we have only measured the thyroid hormones, but the other hormones that affect both thyroid function and energy expenditure, such as leptin, may play important roles.

In summary, our data support a model in which RMR scales positively with PA and that this association occurs alongside measurable variations in thyroid hormone levels. However, the contribution of thyroid hormones to this relationship was small, and their inclusion did not meaningfully alter the association between physical activity and RMR. Notably, we did not observe compensation in RMR in response to elevated PA, and TEE or PAL did not stabilise at a plateau with elevated PA levels as proposed in the constrained model. However, the levels of physical activity in our study population were relatively low, and hence, they may have been performing at a level below that where constraints in total expenditure became apparent. Nevertheless, at these low activity levels, associations between PA and THs were still apparent, even if they did not mediate much of the association between PA and RMR.

## Data Availability

The raw data supporting the conclusions of this article will be made available by the authors, without undue reservation.
