# Supplementary material for: The associations between resting and total energy expenditure, physical activity, and thyroid hormone levels in adult females
Source: Front Physiol. 2026 Mar 31;17:1716140. doi: 10.3389/fphys.2026.1716140 (PMC13076169; doi:10.3389/fphys.2026.1716140)
Supplement: Supplementary file 1 [file DataSheet1.pdf]

Supplementary materials

Supplementary methods

Power analysis

The sample size was calculated using the PASS 2020 (version 20.0.6) software before the study by power analysis. The effect size was calculated using previously published data (Hu et al., 2022). Multiple regression test was used with a power of 0.8, and the alpha with 0.05. FFM, FM and age were set as covariates. Calculated sample size was 37, considering the dropouts, 46 subjects were recruited.

The actual analytical sample was limited to 38 because several individuals did not meet the inclusion criteria or had incomplete data (Figure 1). For transparency, we performed a post-hoc sensitivity analysis in PASS using the predictors retained in the final stepwise model (T3, physical activity, and FFM), $\alpha = 0.05$, and the sample size ($n = 38$). The minimum detectable effect (MDE) for the full 3-predictor model was $R^2 = 0.244$, corresponding to 80% statistical power.

Supplementary Table S1. Shapiro-Wilks test results of variables for normal distribution.

Variable	W statistics	P value
FFM	0.93	0.015
FM	0.94	0.06
Age	0.90	0.003
RMR	0.94	0.04
TEE	0.89	0.001
PA	0.86	0.0003
T3	0.93	0.02
T4	0.98	0.71
fT3	0.97	0.49
fT4	0.98	0.68
TSH	0.79	<0.0001

W Statistic: The value of the Shapiro-Wilk statistic, FFM; fat-free mass, FM; fat mass, T3; triiodothyronine, T4; thyroxine, fT3; free triiodothyronine, fT4; free thyroxin, TSH; thyroid-stimulating hormone (n = 38).

Supplementary Table 2. Diagnostic Results of the regression models. Predictor of RMR and PA (n = 38).

Stepwise regression model	Shapiro-Wilk Test statistiscts - W	Breusch-Pagan Test – BP	Durbin-Watson Test – d	Variance Inflation Factor - VIF
RMR ~ FFM+FM+Age	0.97, p = 0.47	0.76, p = 0.86	1.92 , p = 0.71	FFM – 4.22, FM – 4.18, Age – 1.02
RMR_residuals ~ THs	0.97, p = 0.32	10.26, p = 0.31	1.97, p = 0.98	T3 - 1
PA ~ THs	0.88, p = 0.001	1.1, p = 0.29	2.22, p = 0.47	T4 - 1
RMR ~ FFM + FM+ PA+ THs	0.98, p = 0.84	2.39, p = 0.49	2.05, p = 0.96	FFM – 1.04, T3 – 1.12, PA – 0.49

The first model is a multiple regression; subsequent models were derived using stepwise regression (n = 38).

Supplementary Table 3. Comparison of Theory-Driven and Stepwise Regression Models: Predictors of RMR.

Model	Significant predictors	Direction	R^2	AIC
Stepwise model (RMR~ FFM+ FM+ PA+ THs)	FFM, T3, PA	+	0.774	59.55
Model 1 (RMR ~ FFM+FM+Age)	FFM, FM	+	0.708	69.22
Model 2 (RMR ~ FFM+FM+ T3+T4)	FFM, T3	+	0.772	61.84
Model 3 (RMR ~ FFM+FM+PA)	FFM	+	0.715	68.23
Model 4 (RMR ~ FFM+FM + PA + T3)	FFM, T3, PA	+	0.778	60.67

AIC; Akaike Information Criterion. Model 1 to 4 are theory-driven models (n = 38).

Supplementary Table 4. Diagnostic Results of the stepwise regression models. Predictor of TEE.

Stepwise regression model	Shapiro-Wilk Test statisticts - W	Breusch-Pagan Test – BP	Durbin-Watson Test – d	Variance Inflation Factor - VIF
TEE ~ FFM+FM+Age	0.89, p = 0.001	2.79, p = 0.43	1.94 , p = 0.71	FFM – 4.22, FM – 4.18, Age – 1.02
TEE ~ FFM + FM+RMR+ PA+ THs	0.94, p = 0.01	3.98, p = 0.14	2.28, p = 0.36	FFM – 3.23, RMR – 3.23

The first model is a multiple regression, and the second model was derived using stepwise regression (n = 38).

Supplementary Table 5. Comparison of Theory-Driven and Stepwise Regression Models: Predictors of TEE.

Model	Significant predictors	Direction	R^2	AIC
Stepwise model (TEE~ FFM+ FM+ PA+ THs)	FFM, RMR	+	0.71	121.26
Model 1_TEE (TEE ~ FFM+FM+Age)	FFM	+	0.66	129.09
Model 2_TEE (TEE ~ FFM+FM+RMR)	FFM, RMR	+	0.71	123.12
Model 3_TEE (TEE~ FFM+FM+PA)	FFM	+	0.68	127.55
Model 4_TEE (TEE~ FFM+FM+T3+T4)	FFM, T3	+	0.71	125.87
Model 5_TEE (TEE~ FFM+FM+RMR+PA+T3)	FFM	+	0.75	122.19

AIC; Akaike Information Criterion. Model 1_TEE to 5_TEE are theory-driven models (n = 38).

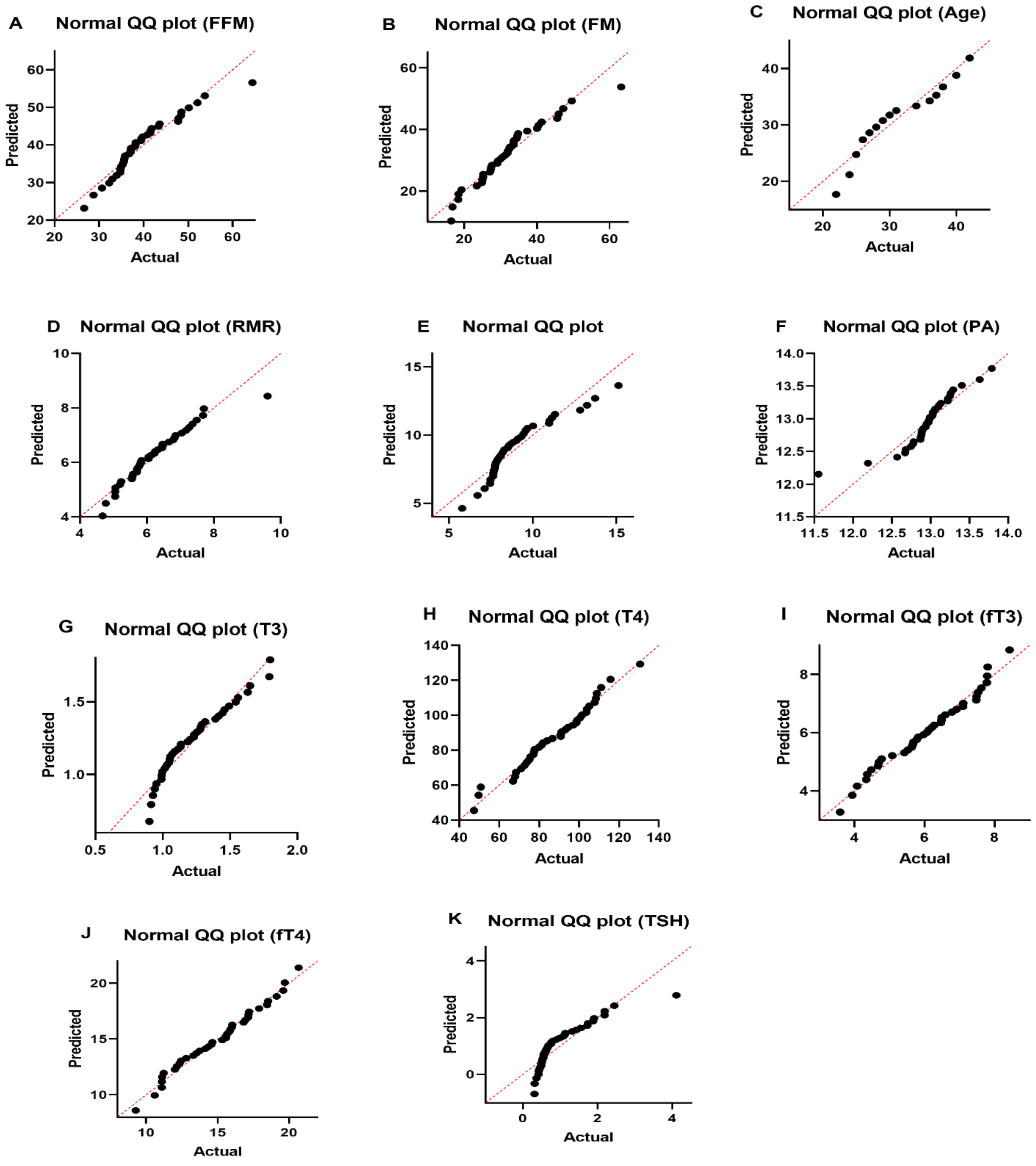

Supplementary figure 1 QQ plots of the variables (n = 38)

A- QQ plot of FFM, B – QQ plot of FM, C - QQ plot of Age, D - QQ plot of RMR, E - QQ plot of TEE, F - QQ plot of PA, G - QQ plot of T3, H - QQ plot of T4, I - QQ plot of fT3, J - QQ plot of fT4, K - QQ plot of TSH.

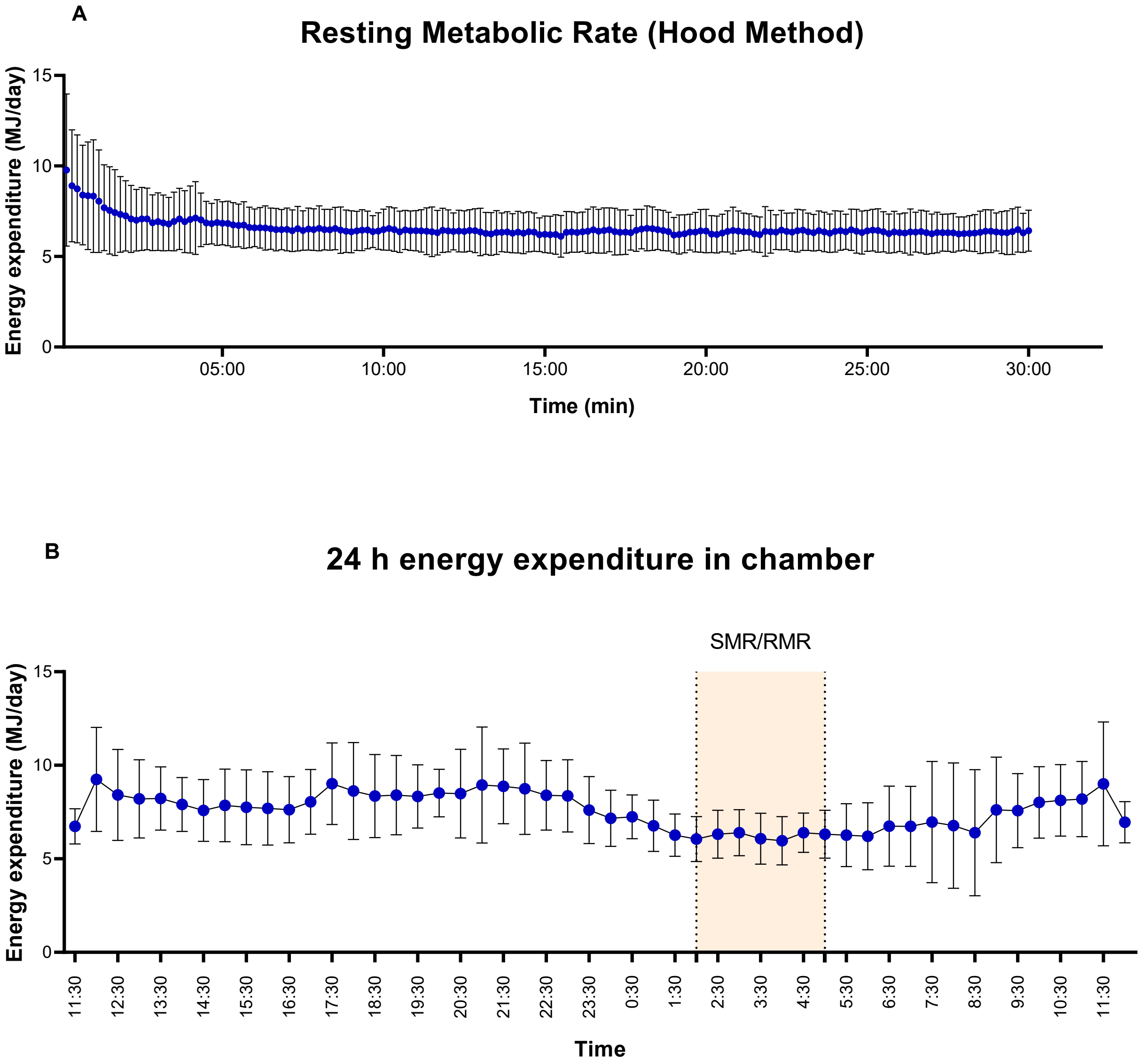

Supplementary Figure 2. RMR measurements from the Hood method and the Chamber

A- The variation of the resting metabolic rate measured from the hood method (n =39). Time is mentioned in minutes, B - 24-hour energy expenditure of subjects in the chamber (n = 33). Time is mentioned in 30-minute intervals. The shaded area represents the lowest and most stable energy expenditure, considered as the subjects' resting metabolic rate. RMR; Resting metabolic rate, SMR; Sleeping metabolic rate.

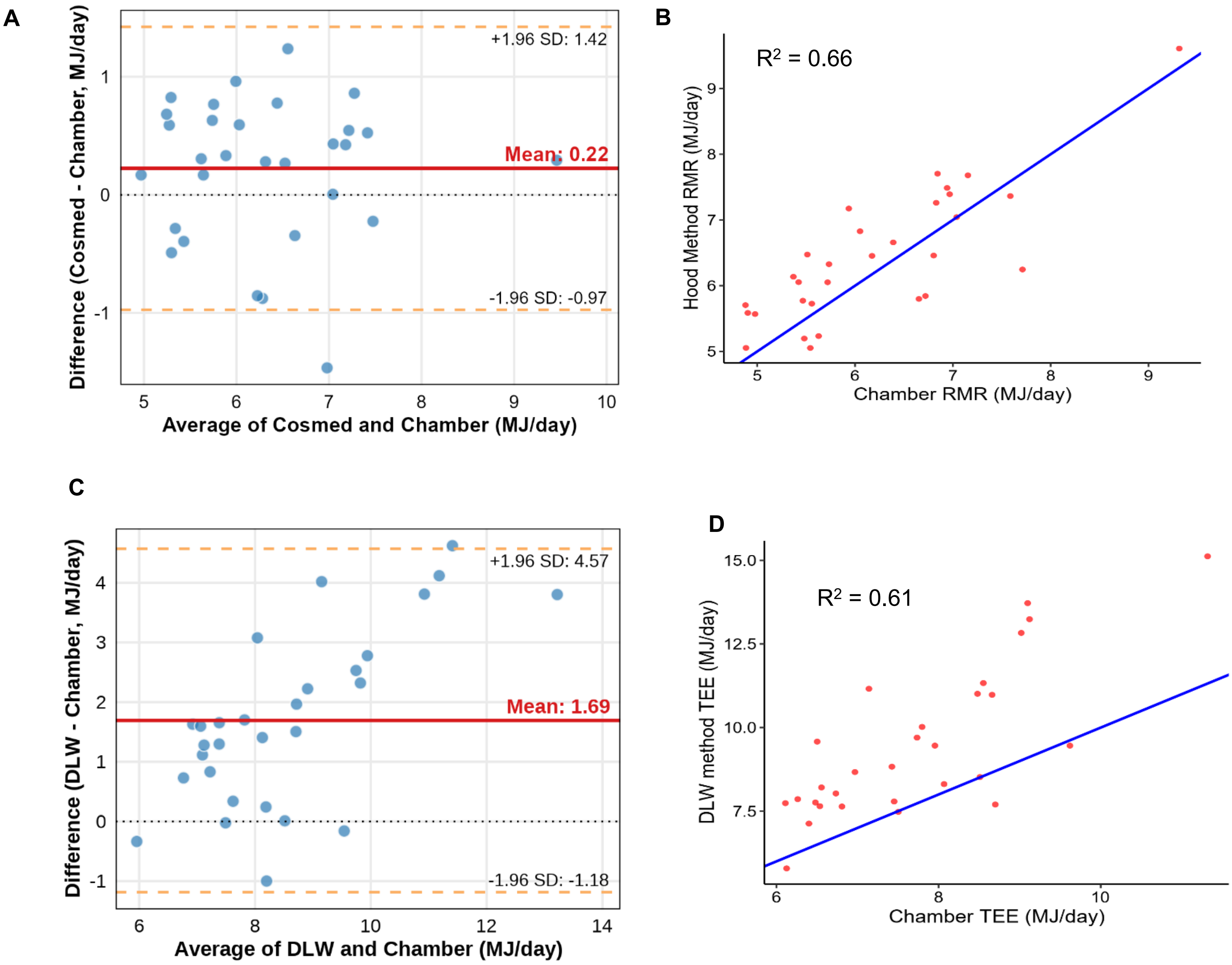

Supplementary Figure 3. A-Bland_Altman plot Hood method and Chamber, S3B-Reduced major axis plot (Hood method and Chamber RMR), C-Bland_Altman plot DLW method and Chamber, D-Reduced major axis plot (DLW method and chamber TEE)

A - Bland Altman plot assessing the agreement between hood and chamber calorimeter methods. The mean bias is 0.22, and the confidence interval is between 1.42 and -0.97 (n = 30). B - Reduced major axis plot shows a higher agreement between the measured sleeping metabolic rate and resting metabolic rate (RMR) ($R^2 = 0.66$) between hood and chamber calorimetry, C - Bland Altman plot assessing the agreement between doubly labeled water (DLW) and chamber calorimeter method. DLW gave higher estimated total energy expenditure (TEE), D - Reduced major axis plot shows a strong linear agreement ($R^2 = 0.61$) between DLW and chamber TEE measurements. All the measurements are presented in MJ/day.

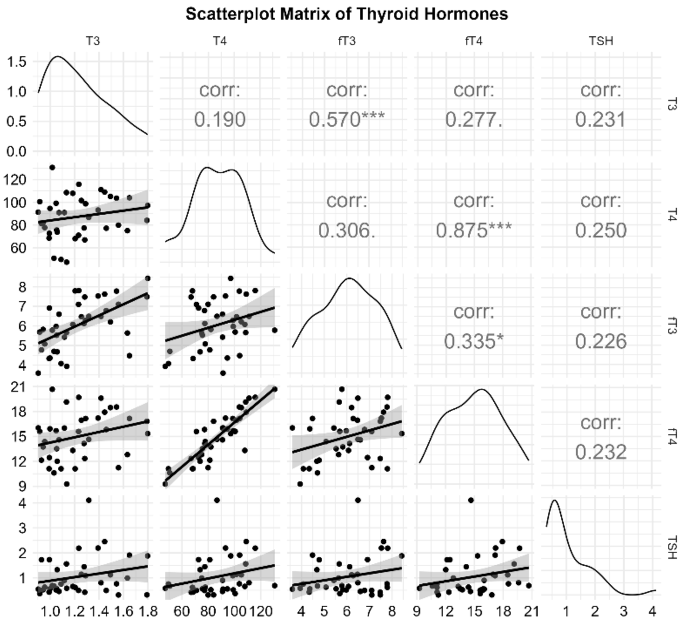

Supplementary Figure 4. Thyroid hormone correlation.

Correlation is present as Spearman's r values. T3; triiodothyronine, T4; thyroxine, fT3; free triiodothyronine, fT4; free thyroxin, TSH; thyroid stimulating hormone (n = 38).

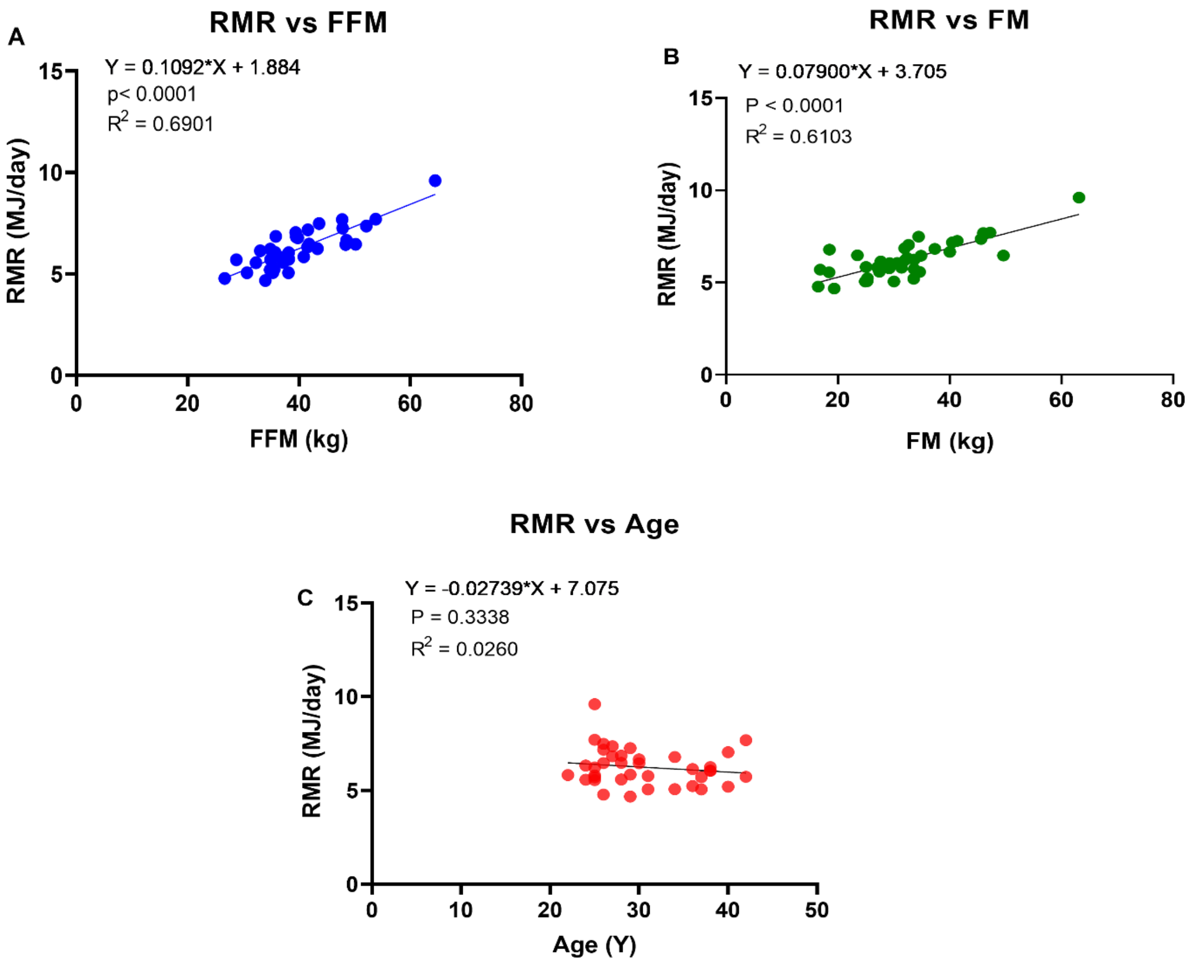

Supplementary Figure 5. Relationship between RMR, body composition and age

A - Association between RMR and FFM, B - association between RMR and FM, and C - association between RMR and age. RMR; resting metabolic rate, FFM; fat-free mass, FM; fat mass (n= 38).

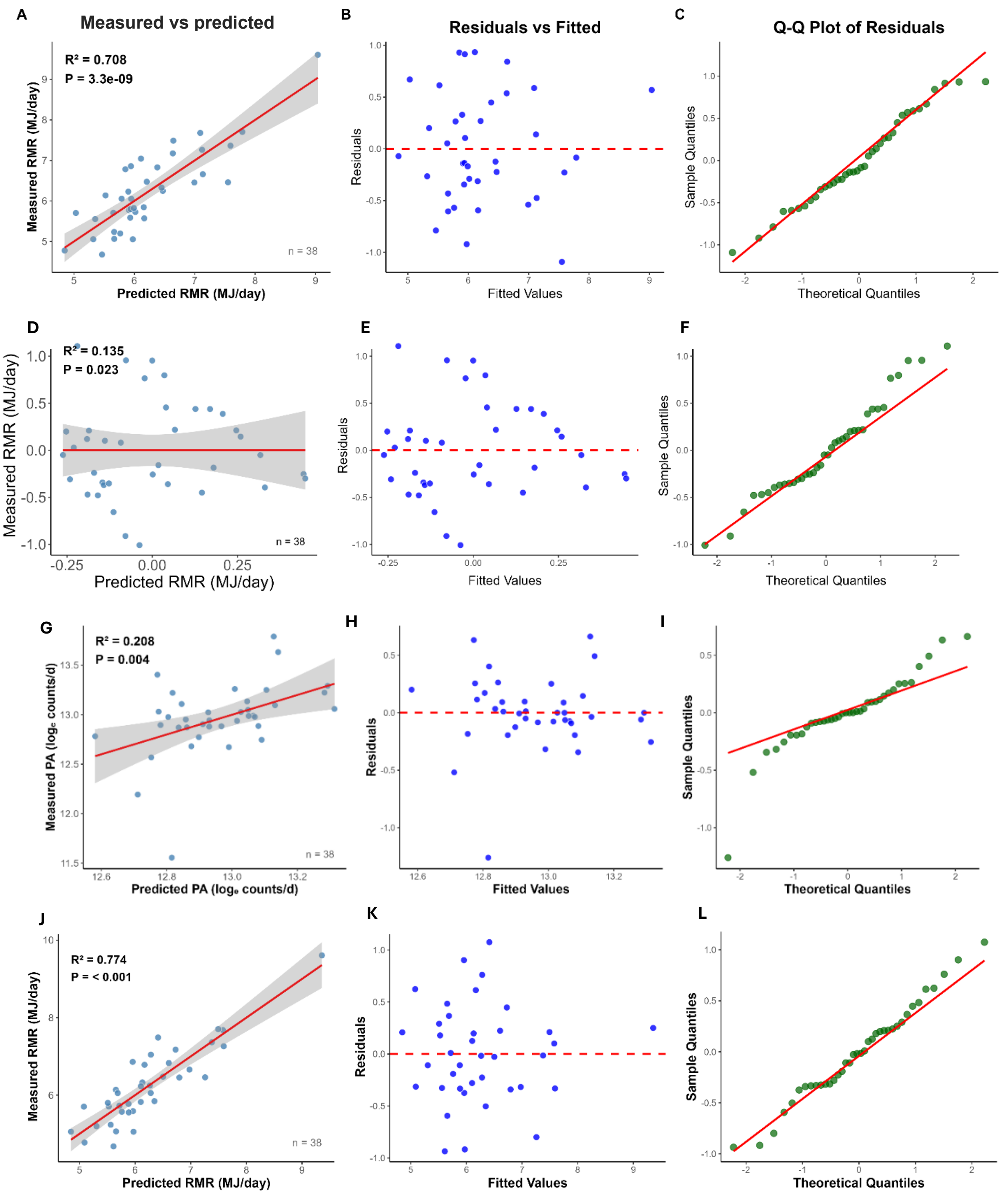

Supplementary figure 6. Stepwise regression model diagnostic plots; predictors of RMR.

Panels A–L show diagnostic checks for the four stepwise regression models used to identify significant predictors of RMR. Each model includes three plots: predicted vs. observed values (fit assessment), residuals vs. fitted values (homoscedasticity and linearity check), and normal Q–Q plots (residual normality). Panels **A–C:** RMR ~ FFM+FM+Age, Panels **D–F:** RMR_residuals ~ THs, Panels **G–I:** PA ~ THs, Panels **J–L:** RMR~ FFM+FM+PA+THs (n = 38).

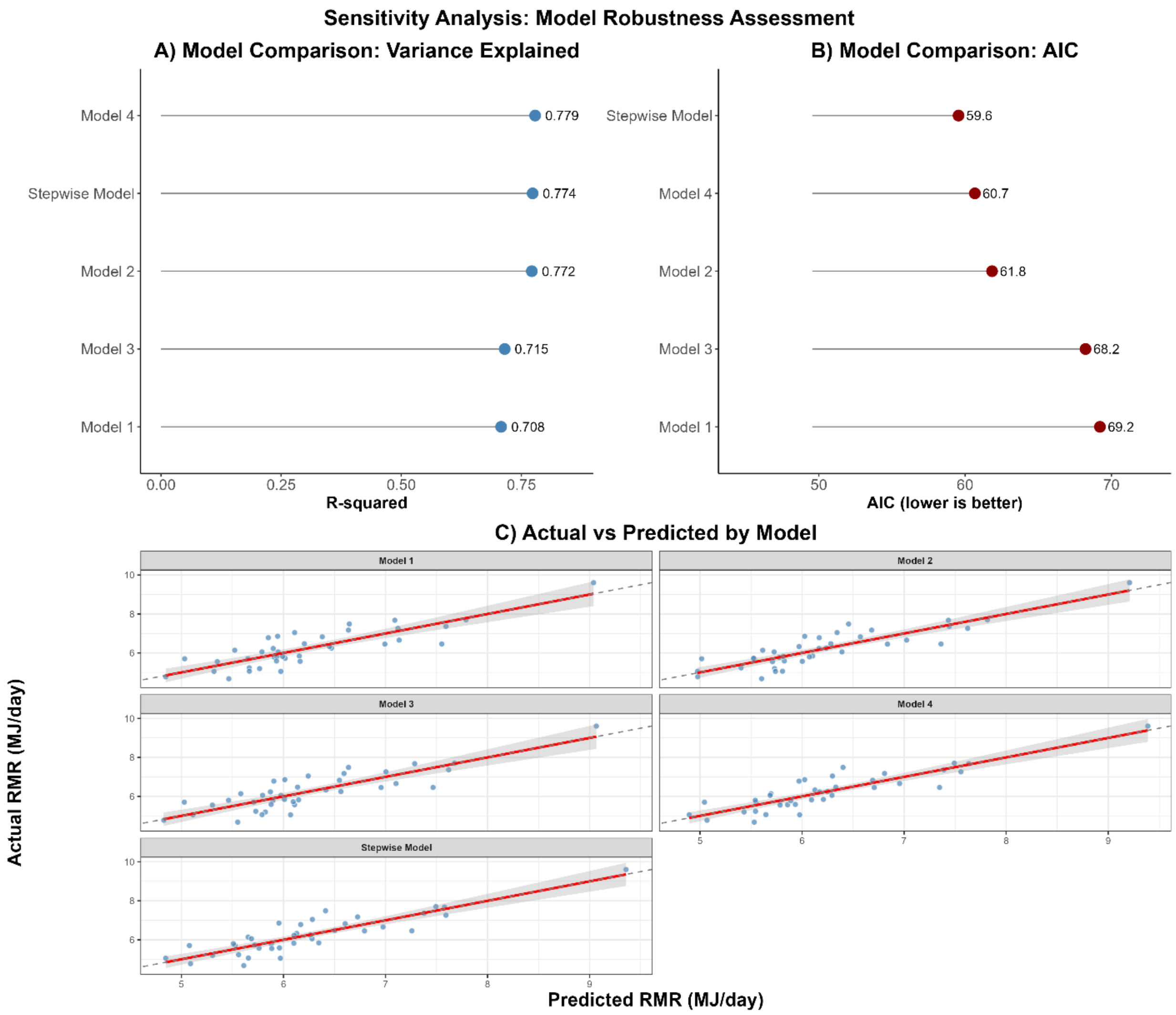

Supplementary figure 7. Sensitivity analysis of the theory-driven and final stepwise regression models.

This figure shows the sensitivity analysis results for both the theory-derived model (Model 1-4) and the final stepwise model, illustrating how model estimates change when key predictors are systematically varied. For each model, R^2 and Akaike Information Criterion (AIC) values are presented for the robustness of associations between physical activity, thyroid hormones, and resting metabolic rate (n = 38).

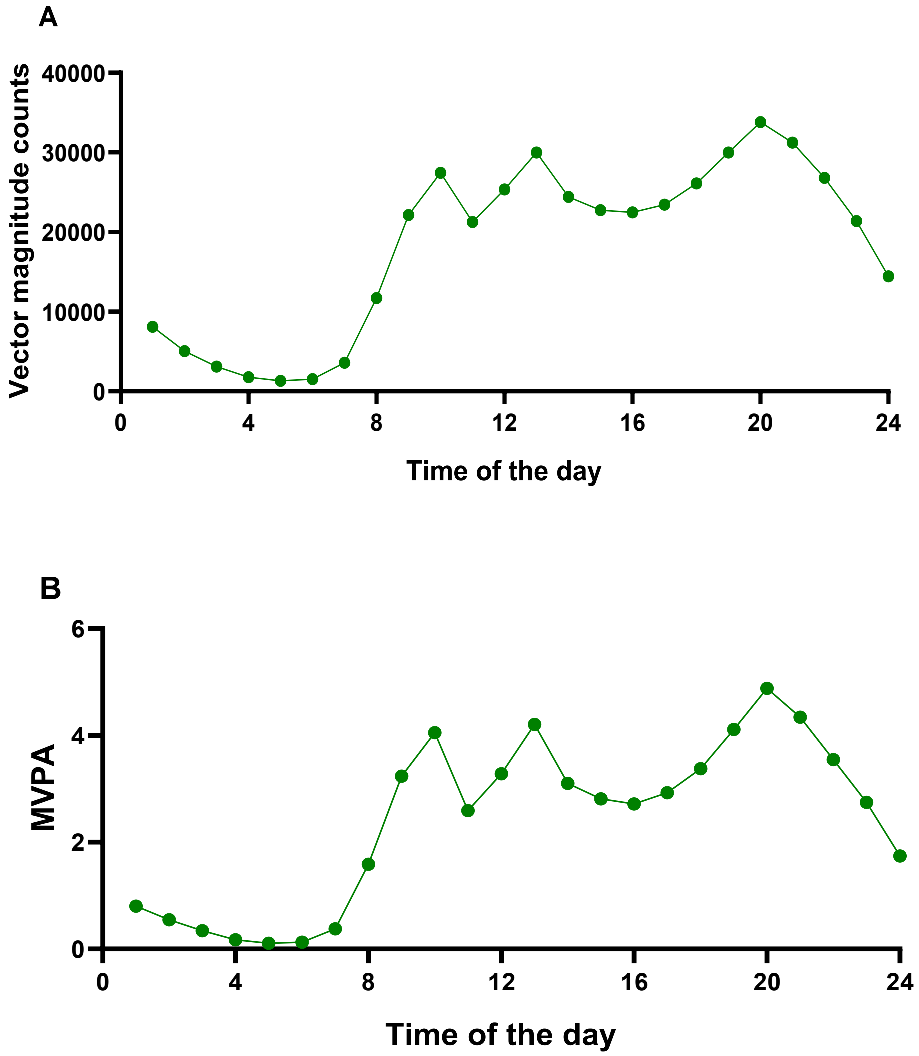

Supplementary figure 8. Variation of daily activities

Represents the physical activity level across the 24-hours of a day. A- Vector magnitude counts in a day. B - represents the moderate to vigorous physical activity level across the 24 hours of a day. MVPA; moderate to vigorous physical activity (n = 38).

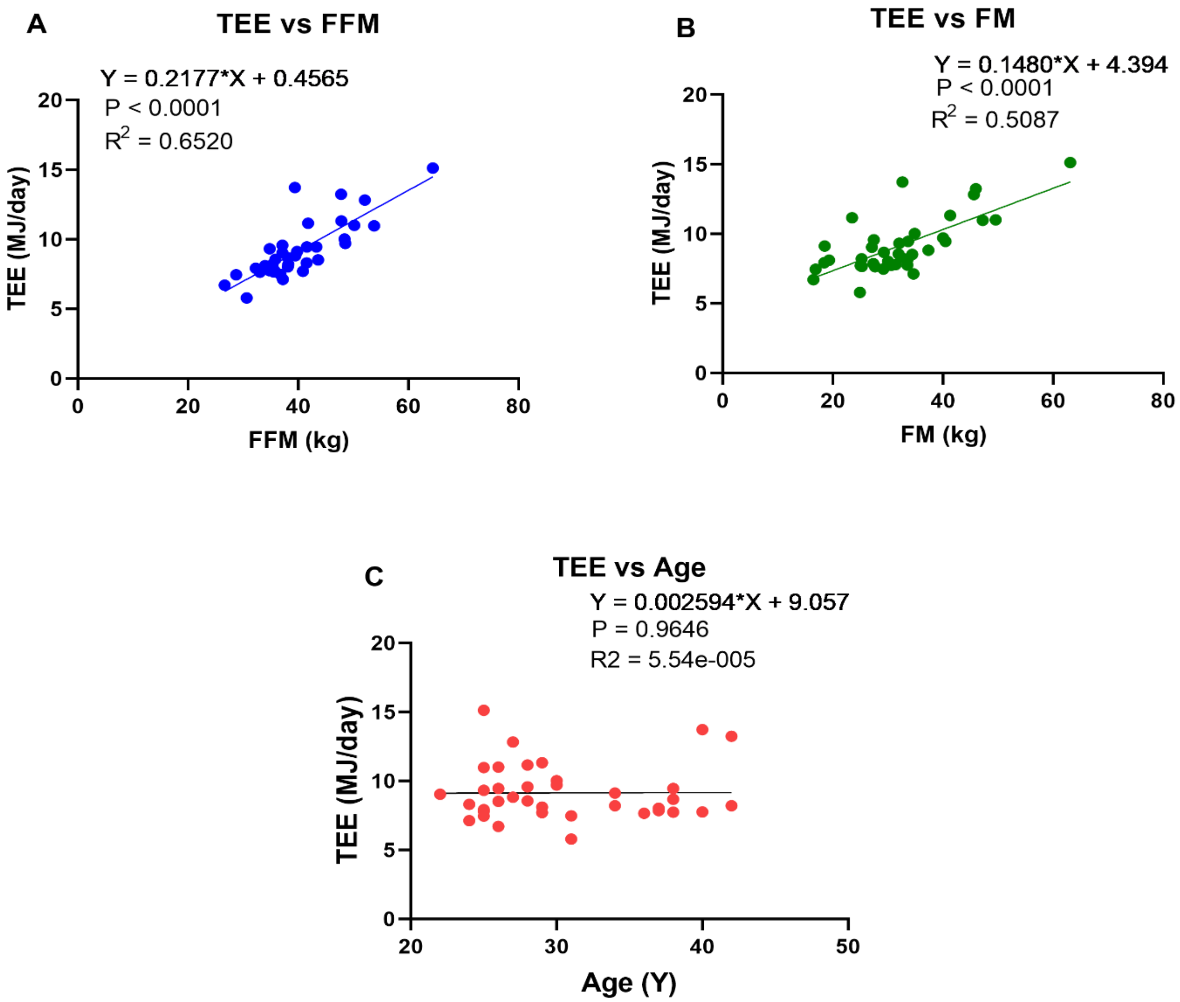

Supplementary Figure 9. Relationship between TEE, body composition and age

A - Association between TEE and FFM, B - association between TEE and FM, and C - association between TEE and age. RMR; resting metabolic rate, FFM; fat-free mass, FM; fat mass, all the TEE measured from Doubly labelled water method (n = 38).

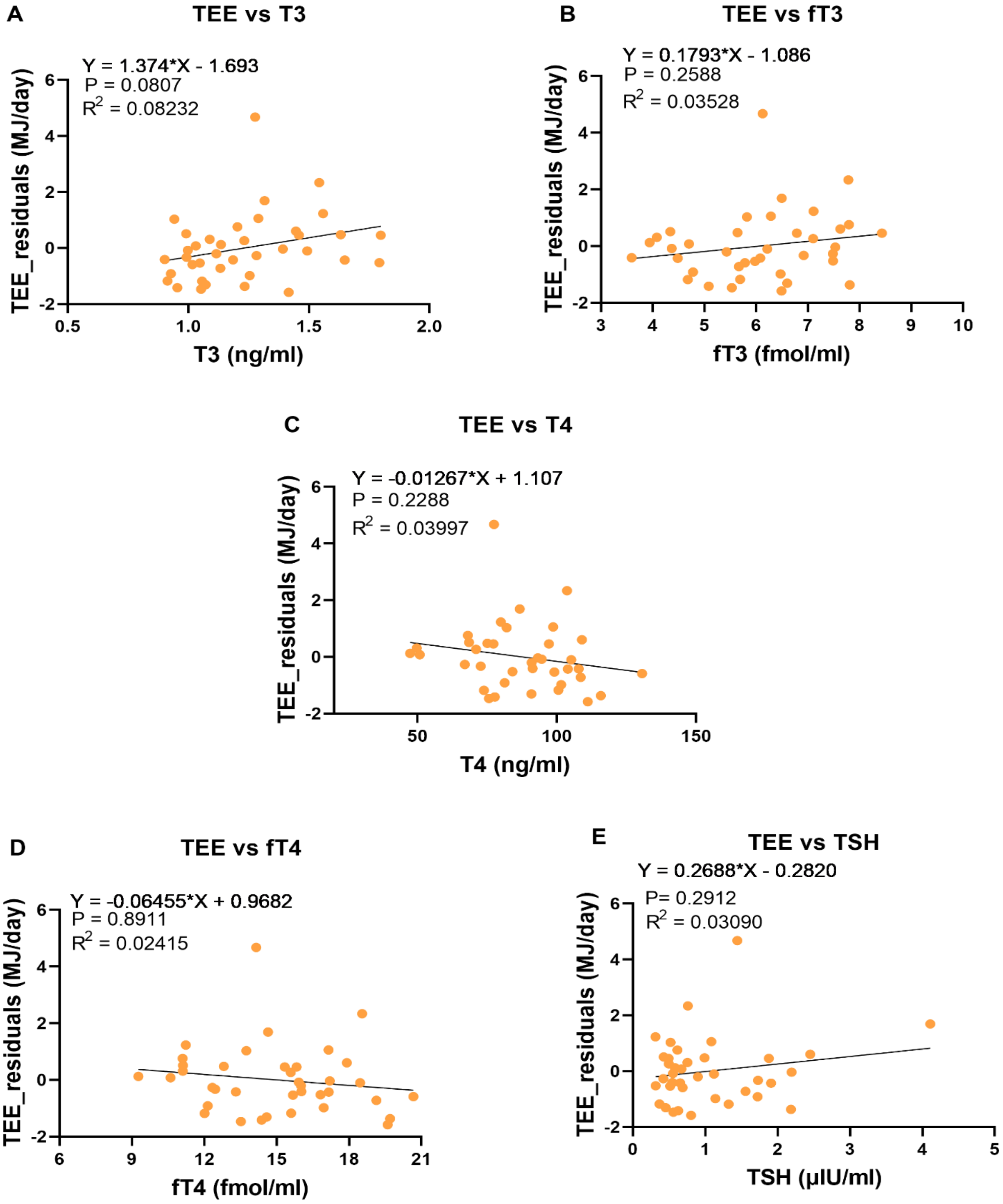

Supplementary Figure 10. Relationship between residual TEE and TH.

Association between thyroid hormone and the total energy expenditure adjusted for the fat-free mass (n = 38). The TEE measured using the doubly labelled water method was included. A-association between T3 and TEE, B - Association between T4 and TEE, C- association between fT3 and TEE, D- association between fT4 and TEE, E - association between TSH and TEE. T3; triiodothyronine, T4; thyroxine, fT3; free triiodothyronine, fT4; free thyroxin, TSH; thyroid stimulating hormone, TEE; total energy expenditure (MJ/day).

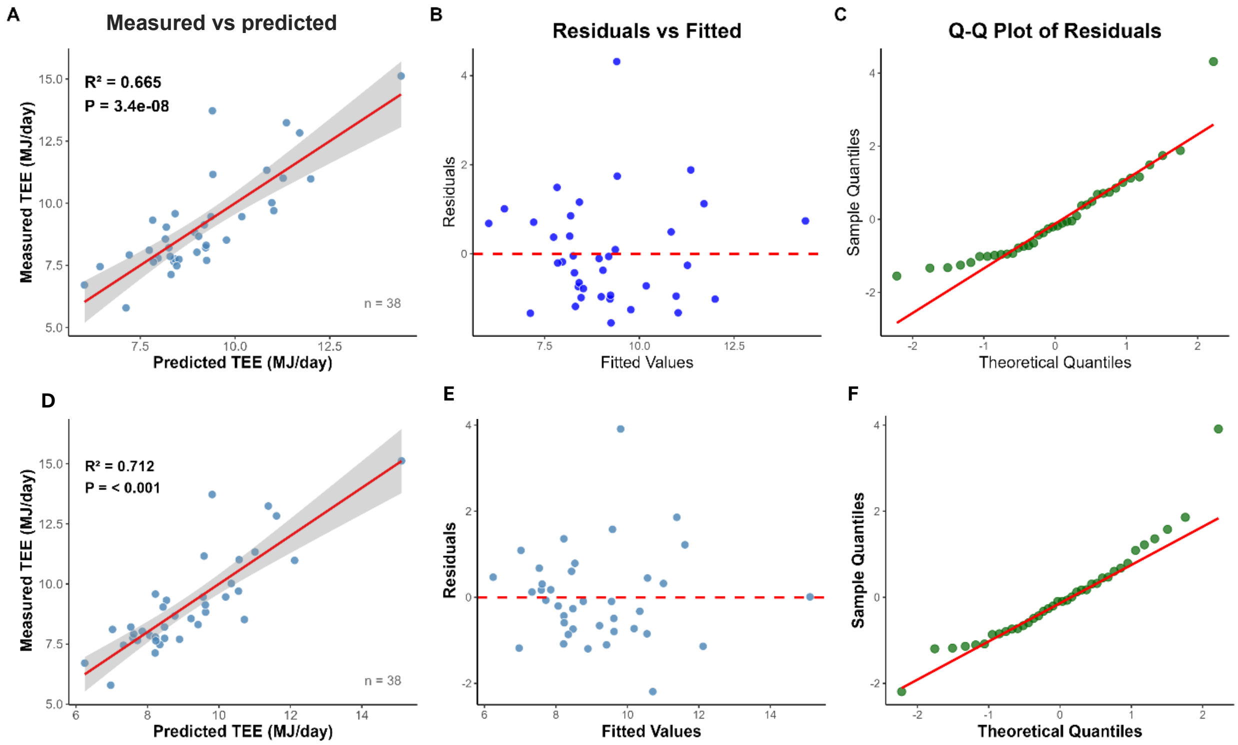

Supplementary figure 11. Stepwise regression model diagnostic plots; predictors of TEE.

Panels A–F show diagnostic checks for the four stepwise regression models used to identify significant predictors of TEE. Each model includes three plots: predicted vs. observed values (fit assessment), residuals vs. fitted values (homoscedasticity and linearity check), and normal Q–Q plots (residual normality). Panels **A–C:** TEE ~ FFM+FM+Age, Panels **D–F:** TEE ~ FFM+FM+RMR+PA+THs (n = 38).

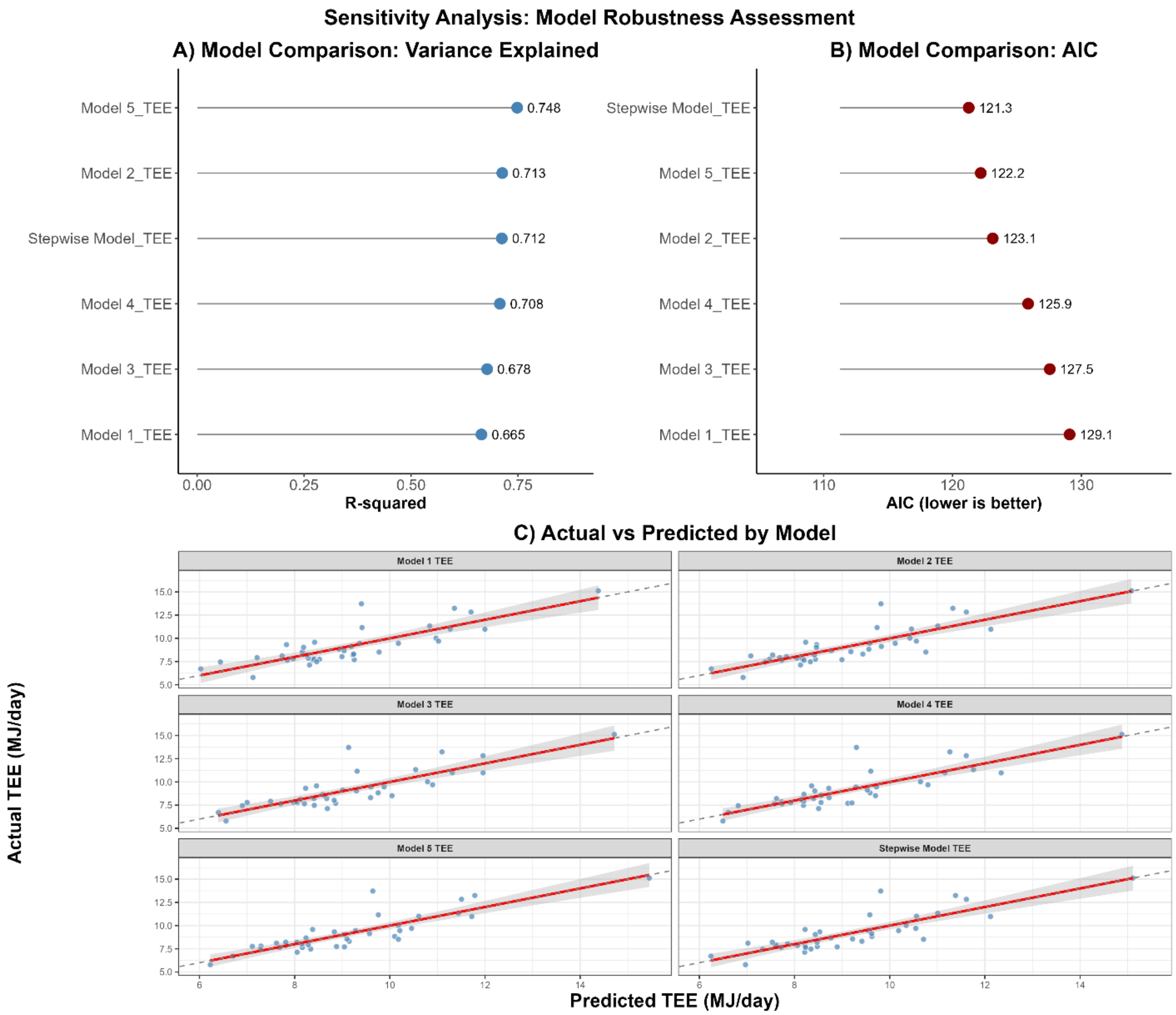

Supplementary figure 12. Sensitivity analysis of the theory-driven and final stepwise regression models.

This figure shows the sensitivity analysis results for both the theory-derived model (Model 1_TEE-5_TEE) and the final stepwise model, illustrating how model estimates change when key predictors are systematically varied. For each model, R^2 and AIC values are presented for the robustness of associations between physical activity, thyroid hormones, resting metabolic rate and total energy expenditure (n = 38).

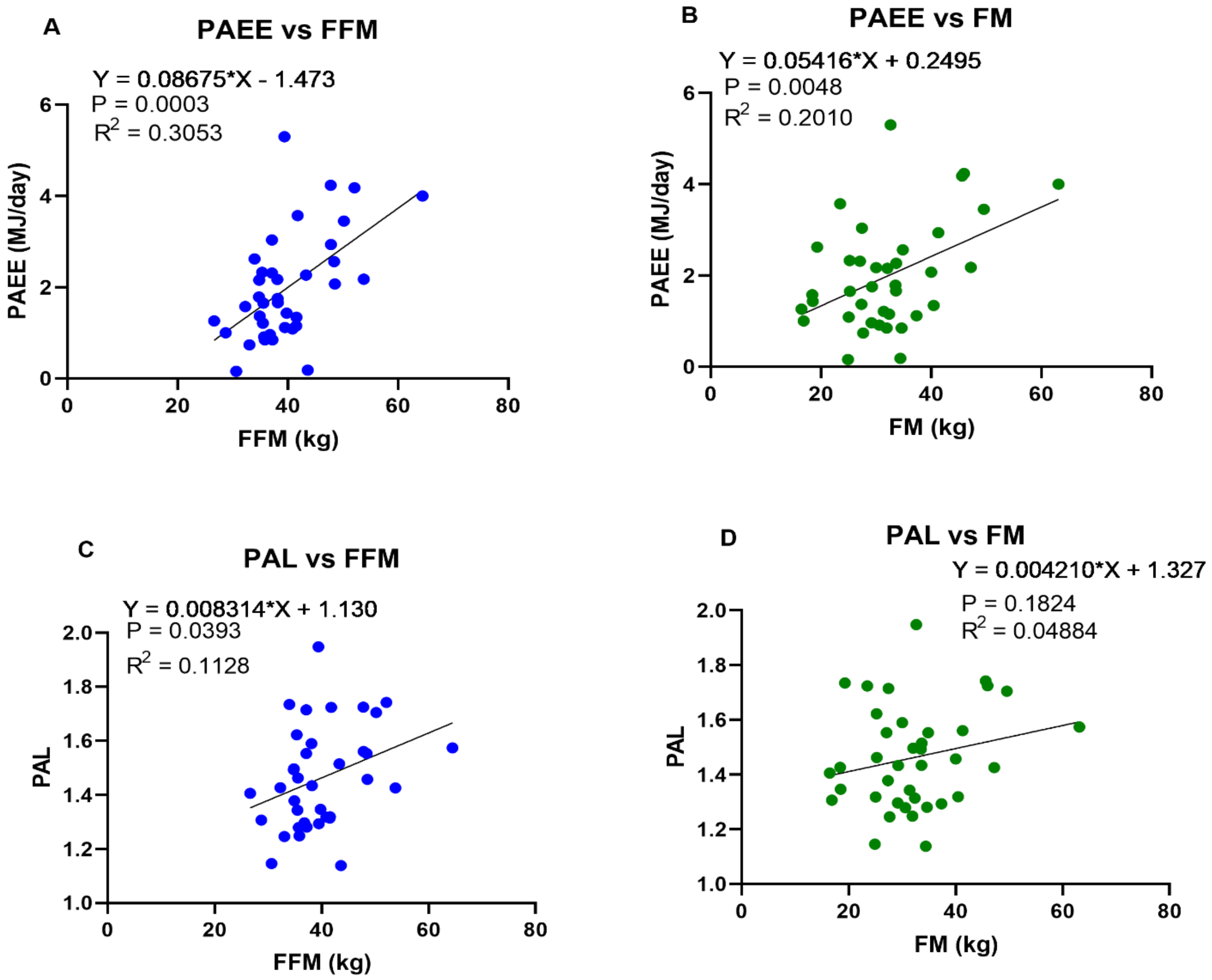

Supplementary Figure 13. Relationship between PAEE, PAL and body composition

A - Association between PAEE and FFM, B - association between PAEE and FM, C – Association between PAL and FFM, D – Association between PAL and FM. PAEE and PAL were calculated from DLW data, while FFM and FM were derived from body composition analysis PAEE; physical activity energy expenditure, PAL; physical activity level, FFM; fat-free mass, FM; fat mass (n =38).
